# Exposure to lysed bacteria can promote or inhibit growth of neighboring live bacteria depending on local abiotic conditions

**DOI:** 10.1093/femsec/fiac011

**Published:** 2022-02-09

**Authors:** Fokko Smakman, Alex R Hall

**Affiliations:** Institute of Integrative Biology, Department of Environmental Systems Science, ETH Zürich, Universitätstrasse 16, 8092 Zürich, Switzerland; Institute of Integrative Biology, Department of Environmental Systems Science, ETH Zürich, Universitätstrasse 16, 8092 Zürich, Switzerland

**Keywords:** microbiology, microbial ecology, ecology, cell death

## Abstract

Microbial death is extremely common in nature, yet the ecological role of dead bacteria is unclear. Dead cells are assumed to provide nutrients to surrounding microbes, but may also affect them in other ways. We found that adding lysate prepared from dead bacteria to cultures of *Escherichia coli* in nutrient-rich conditions suppressed their final population density. This is in stark contrast with the notion that the primary role of dead cells is nutritional, although we also observed this type of effect when we added dead bacteria to cultures that were not supplied with other nutrients. We only observed the growth-suppressive effect of our dead-bacteria treatment after they had undergone significant lysis, suggesting a key role for cellular contents released during lysis. Transcriptomic analysis indicated changes in gene expression in response to dead cells in growing populations, particularly in genes involved in motility. This was supported by experiments with genetic knockouts and copy-number manipulation. Because lysis is commonplace in natural and clinical settings, the growth-suppressive effect of dead cells we describe here may be a widespread and previously unrecognized constraint on bacterial population growth.

## Teaser

Dead bacterial cells can both support and inhibit growth of other bacteria around them based on how they died, and the local abiotic environment.

## Introduction

In any natural environment, microorganisms face environmental stressors that can kill them. These include abiotic factors like temperature, nutrient or pH stress, and biotic factors such as infectious viruses (bacteriophages) and antibiotics produced by other microorganisms. Because cell death is expected to release compounds such as amino acids (Zinser and Kolter 1999), it potentially enhances growth of surrounding cells (Corchero *et al*. 2001, Takano *et al*. 2017). Indeed, exposure to dead cells has been shown to support growth of neighboring live bacteria (Steinhaus and Birkeland 1939, Nioh and Furusaka 1968) and is thought to play a key role in population survival of bacteria during extended periods after other nutrients are exhausted (Zambrano and Kolter 1996, Zinser and Kolter 1999, 2004, Takano *et al*. 2017, Schink *et al*. 2019). However, dead cells and associated debris may also induce other types of changes in gene expression in living cells through the release of, for example, Extracellular DNA (eDNA) (Jakubovics *et al*. 2013), membrane vesicles (Schwechheimer and Kuehn 2015, Turnbull *et al*. 2016) or signaling molecules (LeRoux *et al*. 2015b, Westhoff *et al*. 2017). For instance, in *Pseudomonas aeruginosa*, cells lysed by a competing strain cause neighboring live cells to upregulate a number of genes involved in antibacterial activity, improving their fitness in interspecific competition (LeRoux *et al*. 2015a). It was recently shown that dead cells can alter swarming behavior in *Escherichia coli* and induce changes in gene expression that benefit swarming *E. coli* when challenged with antibiotics (Bhattacharyya *et al*. 2020). These findings suggest that the ecological role of dead cells may not be limited to nutrition (recycling nutrients and other compounds beneficial for growth).

Much research on the ecological role of dead cells has focused on experimental populations of bacteria that have entered late stationary phase or death phase in batch culture, and subsequent recycling of dead cells present in such cultures as nutrients (Finkel and Kolter 1999, Zinser and Kolter 1999, Farrell and Finkel 2003, 2004, Finkel 2006, Takano *et al*. 2017). For example, *E. coli* can grow in the supernatant of starved cultures, and the amount of growth depends on the density of the starved culture (Takano *et al*. 2017). Although such studies reveal the effects of changes in medium composition caused by bacterial growth, starvation and death, they do not tell us about the effects of dead cells themselves, because they are typically filtered out of the medium before the supernatant is added to live cultures. A recent study overcame this limitation by adding UV-killed culture directly to live cultures, demonstrating a nutritional effect consistent with that suggested by supernatant experiments (Schink *et al*. 2019). However, key gaps in our knowledge still remain. Perhaps most importantly, it is unclear whether exposure to dead cells has a similarly nutritious effect in populations that are also supplied with other nutrients (because nutrient cycling effects are typically measured in starved cultures or nutrient-free medium). Bacteria in nature are not always starving, and responses to dead cells may be different when cells have access to other nutrients. Additionally, the impact of dead cells may depend on how they have died. It has been shown that live cells respond differently to dead kin depending on the cause of death in unicellular algae (Durand *et al*. 2011), yeast (Herker *et al*. 2004) and the eukaryotic immune system (Kono and Rock 2008, Medina *et al*. 2020). Bacteria that are not starved may therefore have different effects on living cells. In particular, cell lysis, which is common in nature as a result of bacteriophage infection (Weinbauer 2004), some antibiotics (Lederberg 1957, Yu *et al*. 2015) or type VI secretion systems (Alteri and Mobley 2016), is likely to release a greater fraction of intracellular contents into the environment and may therefore have different effects compared with those observed for starved cultures.

Here, we investigate the effects of various types of dead bacteria and associated cell debris/lysate on population growth of *E. coli*. We do this in both nutrient-rich and nutrient-poor conditions, revealing environment-dependent effects: we show dead bacteria can serve as nutrients when these are scarce but can, surprisingly, negatively affect final population density when other nutrients are supplied and then exhausted. By resuspending cultures in fresh medium immediately before killing them, we disentangle the effects of dead bacteria (cell debris and released compounds) from medium conditioning during prior growth. We do this for cells killed in various ways, such as heat, sonication and phage lysis. We then use transcriptomic analysis and genetic knockouts to show that dead bacteria trigger widespread changes in gene expression in neighboring live cells, indicating a key role of upregulated motility genes in the observed population-level response to dead cells in nutrient-rich conditions. Together, these results suggest that dead bacteria can have qualitatively different effects on neighboring live cells depending on the local abiotic conditions and how they have died.

## Materials and methods

### Organisms and growth conditions

We used various strains of *E. coli* in different experiments (Table S1, Supporting Information), and for most experiments we used two types of growth medium: LB (Lennox lysogeny broth, Sigma Aldrich, St. louis, Missouri) and M9 mineral medium (containing M9 salts [33.7 mM Na_2_HPO_4_, 22.0 mM KH_2_PO_4_, 8.55 mM NaCl and 9.35 mM NH_4_Cl] plus 1 mM MgSO_4_ and 0.3 mM CaCl_2_ (Sigma Aldrich, St. louis, Missouri). For one experiment, we supplemented M9 with 0.2% (w:v) glucose, glycerol or casamino acids (Sigma Aldrich, St. louis, Missouri). For another experiment, we made nutrient-depleted versions of LB by reducing the concentration of tryptone and yeast extract (Sigma Aldrich, St. louis, Missouri). All incubation was performed at 37°C.

### Growth assays

In our first experiments, we measured the response of *E. coli* to dead cells (prepared as described later) by filling wells in 96-well microplates (Greiner, Kremsmünster, Austria) with either 148.5 μL of dead-cell suspension (prepared from stationary phase cultures containing ∼10^9^ CFU/mL; see later) or control medium (prepared as described later), after which we inoculated each well with 1.5 μL of an independent overnight culture grown in the appropriate medium. For several experiments, we used 50 μL of dead-cell suspension with 98.5 μL of fresh medium instead; this is indicated where relevant in the main text. For all experiments, we inoculated 4–12 replicate populations (depending on the experiment). Starting optical densities (ODs) were measured using a spectrophotometer (Tecan NanoQuant Infinite M200 Pro, Tecan, Männedorf, Switzerland), after which the microplates were incubated for 24 h, before measuring their final densities. We then calculated the change in OD600 over 24 h for each well (∆OD = OD600*_t_*_=__24 h_ − OD600*_t_*_=__0 h_).

For some experiments, we used a second measure of bacterial growth by counting colony-forming units (CFU/mL), after plating dilution series onto LB agar plates. We used Welch's *t*-test to determine whether growth yield differed on average between cultures treated with the dead-cell suspension and the control treatment.

### Preparation of dead cells

We tested the effects of various types of dead bacteria and corresponding control treatments. In every experiment, we confirmed that dead-cell aliquots contained no viable CFUs by plating 50 μL onto LB agar and incubating overnight. Additionally, we prepared dead bacteria from a marked version of the ancestral strain, K-12 MG1655 Δ*Ara*, allowing us to test for regrowth from killed cultures during the assay by plating on tetrazolium agar (Lenski *et al*. 1991). We did not observe any Δ*Ara* colonies in any of our assays. Dead cells were always made from overnight cultures at stationary phase (∼10^9^ CFU/mL). We made dead-cell suspensions in the following ways:

- Heat-killed cells. We centrifuged overnight cultures of *E. coli* at 4000 rpm for 5 min, after which we resuspended the cultures in the appropriate growth medium. This procedure removes all medium conditioning by prior growth and allows us to investigate the effect of dead cells directly. We then heated these cultures at 100°C for 1 h. As a control, we used 150 μL aliquots of sterile medium subjected to the exact same procedure.
- Sonicated cell lysate. We sonicated 5 mL cultures for 5 min with 1 s pulses at 80% amplitude using a SonoPlus HD2070 (Bandelin, Berlin, Germany). Prior to sonication, we resuspended cultures in fresh medium. Plating showed that sonication did not kill 100% of the population, so we then filtered (0.20 µm) each lysate after sonication. All culture tubes were kept on ice between resuspension and sonication/filtration. As above, the control treatment was sterile medium subjected to the same procedure
- Phage-lysed cells. We hypothesized that lysis by bacteriophages would produce similar effects compared with lysis by sonication. We diluted overnight cultures of *E. coli* 100-fold into 5 mL fresh LB and grew them for ∼2.5 h before resuspending in fresh LB. Then, we added ∼6 × 10^9^ plaque-forming units of lytic phage T7 suspended in 100 μL and further incubated until lysis was visible (∼3 h). We filtered the lysate (0.20-μm filter) to remove any viable cells (confirmed by plating on LB agar). Note this procedure does not remove the phages. To ensure this did not influence our subsequent assays, as the live *E. coli* here we used a strain (ECOR9, from the ECOR collection; Ochman and Selander 1984) that is closely related to *E. coli* K-12 but fully resistant to this phage. To control for the possibility that ECOR9 would react differently to dead-cell lysate compared with *E. coli* K-12, we also tested ECOR9 with dead-cell lysate produced by sonication and compared this with K-12 under the same treatment.

### RNA isolation and sequencing

To test for changes in gene expression upon exposure to dead cells, we extracted total RNA from *E. coli* exposed to sonicated *E. coli* or the corresponding control treatment (three replicates each) at three different time points (after 5, 6.5 and 24 h of growth). We grew *E. coli* the same way as in our growth assays described earlier, adding 50 µL of sonicated and filtered dead cells or control medium. We used 50 µL here, rather than 148.5 µL as in some of our experiments, to minimize the amount of extracellular RNA in the medium that was not from our focal culture, while still producing a clear effect of dead cells on growth. Additionally, cultures were pelleted and resuspended before RNA extraction, which we expect to remove the majority of any free-floating RNA derived from the lysate rather than contained in live cells. To obtain sufficient culture volume for RNA extraction, for each replicate we pooled six separate wells inoculated from the same overnight culture and incubated in the same conditions (we did this, rather than simply incubating larger culture volumes, to keep the culture volume and growth conditions identical to the main experiments earlier). We then pelleted the pooled cultures, removed supernatant, and added Qiagen RNAprotect Bacteria Reagent. We then extracted total RNA with the SV Total RNA isolation kit. Total RNA was then DNase treated using the Ambion Turbo DNA-Free DNase kit (Life technologies), and ribosomal RNA was removed using the MICROBExpress bacterial mRNA enrichment kit (Thermo Fisher). Resulting read-alignment was performed with bowtie2 (Langmead and Salzberg 2012) using the Ensembl genome build K-12_MG1655_ASM584v2 as reference. Gene expression values were computed with the function featureCounts from the R package Rsubread (Liao *et al*. 2013) (using the following settings: min mapping quality 10, min feature overlap 10 bp, count multi-mapping reads, count only primary alignments, count reads also if they overlap multiple genes). We computed differential expression using the generalized linear model implemented in the Bioconductor package DESeq2 (Love *et al*. 2014). Genes were considered expressed if they had at least 10 reads assigned.

### Motility assays

To test the effect of dead cells on motility of *E. coli*, we used 0.2% Tryptone agar (1% Tryptone, 0.5% NaCl, 0.2% agar) plates that allow bacterial swimming (Kearns 2010). Because *E. coli* K-12 MG1655 is known to be a fastidious swarmer on normal agar due to lacking an O-antigen (Berg 2005), we chose to focus on swimming motility. As negative controls, we used the *fliA* and *flhC* mutants, which are unable to swim (Fig. S1, Supporting Information). We poured plates with 20 mL of Tryptone agar and dried them half-open in a laminar flow hood for 20 min, after which we either (i) spotted 20 μL of dead cells/control medium mixed with 5 μL of overnight culture resuspended in fresh LB in the center of the plate, or (ii) spotted 5 μL of resuspended overnight culture in the center of the plate and 20 μL droplets of dead cells or control medium (LB) 3 cm either side of the inoculation point. We incubated the plates at 37°C wrapped in plastic to maintain humidity. Photographs were taken after 24 h. To analyze the plates, we used ImageJ software (Schneider *et al*. 2012) to measure the maximal distance from the edge of the colony to the edge of the motility halo (maximal radius; Fig. S1, Supporting Information).

### Biofilm formation assay

To test for biofilm formation, we grew *E. coli* as described in our normal growth assays (using a 1:50 live:dead ratio in a total culture volume of 150 μL) and performed a crystal violet assay (Fletcher 1977, Pratt and Kolter 1998, O'Toole *et al*. 1999). We measured OD600 after 24 h without shaking the plate as to not disturb the biofilm, after which we removed the supernatant. We washed all wells once with 150 μL phosphate buffered saline (PBS) to remove planktonic cells, after which we added 175 μL of 0.1% crystal violet (Merck) to stain cells. We incubated the plate for 10 min at room temperature, after which we removed the crystal violet and washed all wells four times with 150 μL PBS to remove excess stain. After removing the final wash, we air-dried the plate for 15 min in a laminar flow hood, after which we added 150 μL of 95% ethanol (EtOH) to solubilize the incorporated stain. We mixed all wells by pipetting up and down. The dye was allowed to solubilize for 10 min with the plate lid closed, after which we measured OD at 590 nm.

### Keio knockout and ASKA overexpression assays

From gene expression data provided earlier, we selected a subset of genes for further investigation. We used the omics dashboard on the EcoCyc website (Paley *et al*. 2017) to identify genes meeting the following criteria: (i) a fold change of >2 at one or more time points, (ii) >10 downstream genes (in the case of regulatory genes) with fold changes of >2 at at least one time point (downstream genes were also identified with the omics dashboard). Of the 330 genes that met at least one of these criteria, we then picked 9 genes for further investigation, prioritizing those with regulatory functions. We then took single-gene knockout mutants of these genes from the Keio collection (Baba *et al*. 2006) and tested their response to dead cells for an altered response compared with the ancestor, using the same protocol as described earlier for sonicated, lysed cells. For two genetic knockouts that showed altered responses to dead cells compared with the wild type (*fliA* and *flhC*), we also tested for an effect of overexpression of the same genes, using strains from the ASKA library (Kitagawa *et al*. 2005). Briefly, these strains each carry a plasmid containing an IPTG-inducible (Isopropyl β-D-1-thiogalactopyranoside) copy of the relevant open reading frame, allowing us to test the effect of overexpression in *E. coli*. We assayed these two strains and MG1655, as described earlier, both with and without 0.1 mM of IPTG to induce the plasmid-borne copies of the gene.

## Results

### Exposure to cell lysate can inhibit or increase population growth depending on abiotic conditions

**Figure 1.**
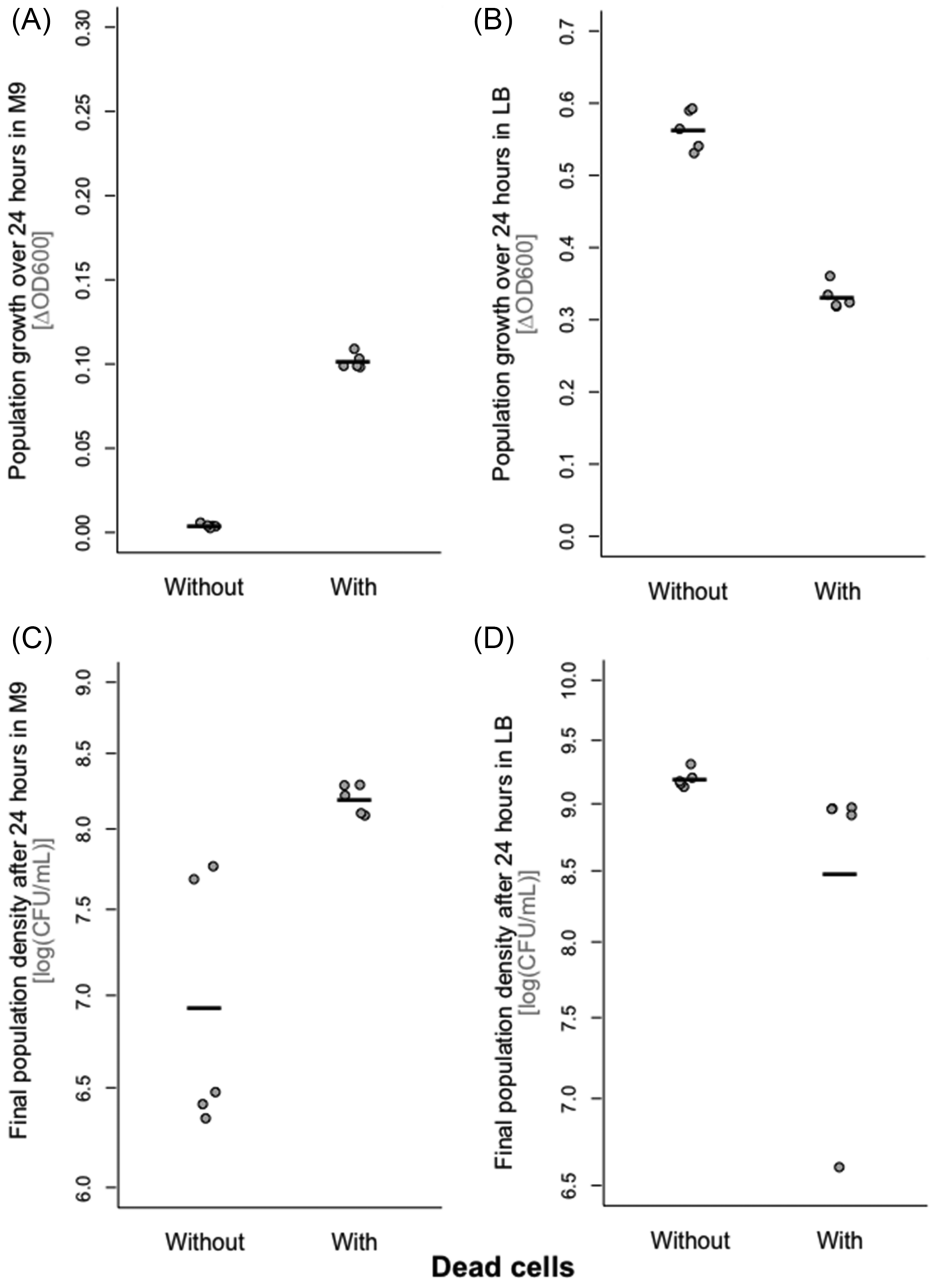
Population growth of *E. coli* K-12 MG1655 in minimal medium (M9 without carbon source; **A**and**C**) and nutrient-rich medium (LB; **B**and**D**), in the presence and absence of dead-cell lysate (*x*-axis; lysate produced by sonication and filtration). Population growth is shown by two different measures: (A and B) change in optical density (OD600) over 24 h and (C and D) CFU/mL of the same cultures. All points represent independent replicates (*n* = 5) and individually prepared cultures of dead cells/controls. Each line shows the mean for one treatment group. We used a total volume of 150 µL, consisting of 1.5 µL overnight culture with 148.5 µL of dead-cell suspension.

To test the effects of dead bacteria, we resuspended cultures of *E. coli* in fresh medium immediately before killing them by sonication, after which we filtered out any surviving cells. Adding this dead-cell preparation (lysate) to live *E. coli* in buffer solution with no other carbon source (M9) supported increased population growth relative to the control treatment: the change in bacterial abundance over 24 h estimated by OD was significantly higher in cultures supplemented with dead-cell lysate than in the control treatment (Welch two-sample *t*-test: *t* = −46.33, df = 4.51, *P* < 0.0001; mean ± SD = 0.004 ± 0.001 without, 0.101 ± 0.004 with dead-cell treatment; Fig. 1A). In contrast, when we added dead cells (lysate) to *E. coli* cultures in nutrient-rich medium (LB), we observed a reduction of final population density compared with cultures without added dead cells (Welch two-sample *t*-test: *t* = 15.79, df = 6.68, *P* < 0.0001; mean ± SD = 0.564 ± 0.028 without, 0.331 ± 0.017 with dead cells; Fig. 1B). We found a similar pattern when we estimated bacterial densities by plating and counting CFUs instead of OD, with an ∼7-fold increase in mean density in M9 (mean ± SD = 2.28 × 10^7^ ± 2.79 × 10^7^ CFU/mL without, 1.60 × 10^8^ ± 3.47 × 10^7^ CFU/mL with dead cells; Fig. 1C) and an ∼2-fold decrease in LB (mean ± SD = 1.58 × 10^9^ ± 2.71 × 10^8^ without, 7.18 × 10^8^ ± 4.02 × 10^8^ with dead cells; Fig. 1D).

In a separate control experiment, we accounted for any possible nutrient depletion in the dead-cell preparation after resuspension and prior to sonication and filtration. We did this by including an additional control treatment where we resuspended *E. coli* as in the dead-cell treatment, but did not sonicate prior to filtration. This showed no effect on both OD and CFU (Fig. S2, Supporting Information), whereas both measures of population growth were negatively affected by addition of sonicated and filtered dead-cell preparation in the same experiment (Fig. S2, Supporting Information). This indicates the negative response we observed in our dead-cell treatments in nutrient-rich medium was not due to nutrient depletion during dead-cell preparation. We also found the magnitude of the response to dead cells was stronger when the initial amount of sonicated cell lysate was higher (148.5 μL instead of 50 μL of killed-cell suspension, with the same total culture volume of 150 μL and live-cell inoculum of 1.5 μL overnight culture; see the ‘Materials and Methods’ section) in both rich medium (dead cells × amount interaction in LB: *F*_1,20_ = 18.93, *P* < 0.001; Fig. S3, Supporting Information) and in buffer (dead cells × amount interaction in M9: *F*_1,20_ = 41.87, *P* < 0.0001; Fig. S2, Supporting Information), although the negative effect in LB was observed for both ratios (Fig. S3, Supporting Information).

When we added cells lysed by bacteriophage T7, instead of by sonication, to cultures of a closely related, T7-resistant *E. coli* strain in nutrient-rich medium, we observed a similar negative effect as earlier for sonicated cells (Welch two-sample *t*-test, *t* = −4.72, df = 3.09, *P* = 0.017; Fig. S4, Supporting Information). This indicates the negative effect of dead cells (lysate) we observed earlier for sonicated cells also applies to cells lysed by natural causes, including bacteriophages.

In contrast, we found cells killed by heat did not support significant growth when added in M9 (Welch two-sample *t*-test, *t* = 2.17, df = 9.96, *P* = 0.055; Fig. S5A, Supporting Information), and had a very minor suppressive effect on OD in LB (Welch two-sample *t*-test for the effect of adding 50 μL dead cells, *t* = 2.39, df = 13.35, *P* = 0.032; Fig. S5B, Supporting Information). Sonicating the cell suspension before heat-treatment restored their negative effect on population growth in LB (Welch two-sample *t*-test: for OD *t* = −12.45, df = 7.25, *P* < 0.0001; for CFU/mL *t* = −12.45, df = 7.25, *P* < 0.0001; Fig. S6, Supporting Information) in LB. This indicates, rather than heat denaturation of cellular contents, it is the lack of sonication and extensive lysis that causes the lack of effect of heat-killed cells. Microscopic observations of different dead-cell suspensions showed those that were only heat-treated contained large clumps of intact, nonviable cells, whereas the phage-lysed suspensions and those that were sonicated before heat-treatment did not (Fig. S7, Supporting Information). Thus, the dead-cell preparations that had the strongest negative effects on population growth of neighboring live cells earlier were those associated with relatively extensive cell lysis and release of intracellular material.

### Adding more nutrients results in stronger growth suppression in response to dead cells

**Figure 2.**
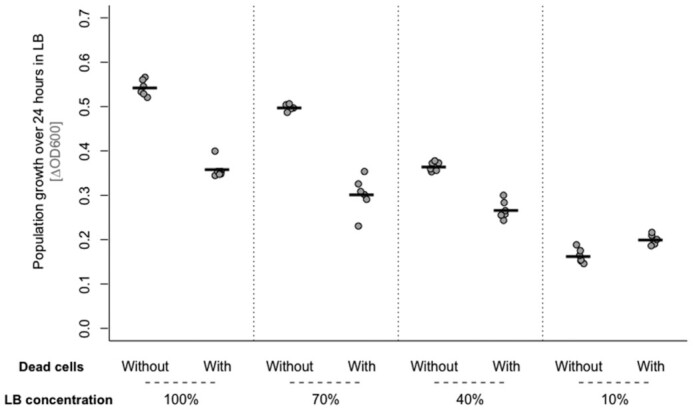
Bacterial population growth (change in OD600 over 24 h) in the presence and absence of dead-cell lysate or control medium, in culture medium with different concentrations of the principal nutrients in LB (tryptone and yeast extract). Lower nutrient concentration treatments were prepared with 5 g/L NaCl, to retain the same osmolarity as in 100% LB. All points represent independent replicates (*n* = 6). Each line shows the mean from one treatment group. We used a total volume of 150 µL in each culture, consisting of 1.5 µL overnight culture with 148.5 µL of dead-cell suspension (produced by sonication and filtration) or control medium.

We hypothesized that the different responses to dead-cell preparations we observed in nutrient-rich medium compared with minimal buffer solution are linked to the different amounts of population growth supported in the two environments. As a first manipulation of this, we tested if the response to dead-cell treatment (lysate) changed when we added a lower amount of nutrients in LB (by using lower concentrations of tryptone and yeast extract, but keeping the salt concentration constant). The negative effect of our dead-cell treatment declined with decreasing nutrient concentration (dead cells × nutrient concentration interaction: *F*_1,44_ = 155.96, *P* < 0.0001; Fig. 2), eventually switching to a positive effect similar to that observed in M9 at the lowest nutrient concentration.

To further investigate how the nutrient environment may influence the response to dead cells, we added dead-cell preparation to cultures growing in minimal buffer supplemented with glucose, glycerol or casamino acids. We observed variable, predominantly positive effects on growth (dead cells × medium interaction: *F*_3,48_ = 6.96, *P* < 0.001; Fig. S8, Supporting Information), similar to those observed in reduced-nutrient-LB treatments supporting equivalent final population densities. Thus, the growth-suppressive effect of exposure to dead-cell lysate on final population density of live cultures was strongest in nutrient-rich conditions supporting a lot of population growth, and we observed some of the same types of dead-cell effects when we used other types of growth media besides LB and M9+glucose.

### Dead cells have similar effects in cultures with different population densities

**Figure 3.**
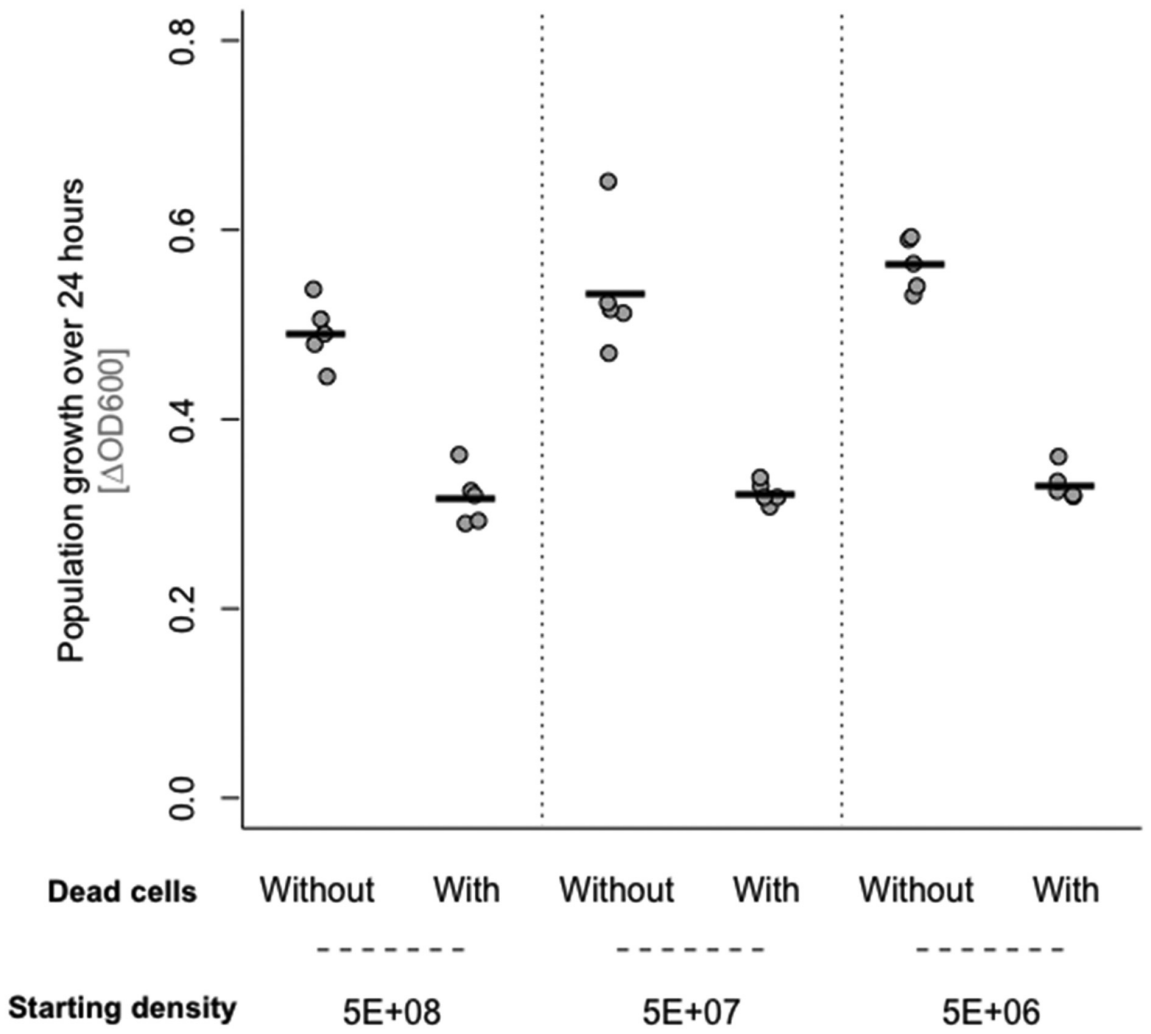
Bacterial population growth (change in OD600 over 24 h) in nutrient-rich medium (LB) in the presence or absence of dead-cell lysate or control medium, using different starting densities of the live cells. In all other assays, 5e+06 was used as the starting density. All points represent independent replicates (*n* = 5). Black lines show the mean. We used a total volume of 150 µL in each culture, consisting of 1.5 µL overnight culture with 148.5 µL of dead-cell suspension (produced by sonication and filtration) or control medium.

The variable effects of dead-cell preparations earlier could potentially be explained by density dependence (exposure to dead bacteria having stronger negative effects per capita when the live population is at higher density, as is reached in higher nutrient LB treatments). This could come about by, for example, interference with density-dependent behaviors such as those mediated by quorum sensing (Bassler 1999, Lazazzera 2000). Alternatively, dead bacteria could inhibit population growth in nutrient-rich medium by reducing the number of new cells produced per unit resource, with a per capita effect independent of the density of the culture. This would be more consistent with mechanisms such as nutrient sequestration or changes in gene expression that affect the efficiency of growth and replication. To disentangle these possibilities, we varied the starting density of the live *E. coli* population. Within the range tested, this did not significantly alter the net change in population density in the absence of dead-cell treatment in rich medium (effect of starting density in LB: for OD *F*_2,12_ = 2.98, *P* = 0.089, Fig. 3; for CFU *F*_2,12_ = 2.18, *P* = 0.16; Fig. S9, Supporting Information). In the presence of dead-cell treatment (lysate produced by sonication and filtration), we observed the same reduced population growth in LB as in our previous experiments earlier, and the magnitude of this effect was independent of starting population density (dead cells × starting density interaction: for OD, *F*_2,24_ = 1.67, *P* = 0.21, Fig. 3; for CFU, *F*_2,24_ = 0.93, *P* = 0.41; Fig. S9, Supporting Information). Thus, while we do not exclude density-dependent effects as constraints on population growth in general, this suggests absolute population density did not explain the variable effects of dead cells we observed earlier in LB. Consistent with this, we also found the positive effect of dead bacteria on population growth in M9 was independent of the starting population density of the live bacteria (dead cells × starting density interaction: for OD *F*_2,24_ = 1.18, *P* = 0.32; for CFU *F*_2,24_ = 0.04, *P* = 0.96; Fig. S10, Supporting Information).

### Exposure to dead bacteria induces changes in gene transcription

**Figure 4.**
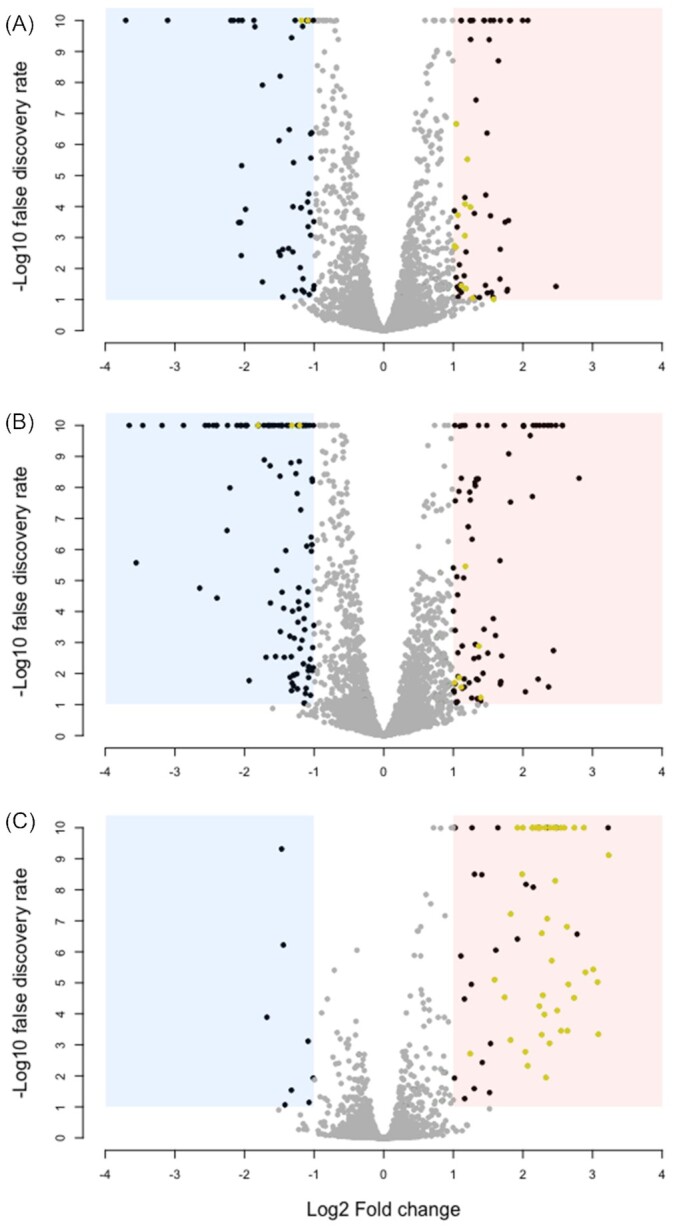
Transcriptional changes of *E. coli* in the presence vs absence of dead-cell lysate. Average gene expression relative to the control treatment is shown after **(A)** 5, **(B)** 6.5 and **(C)** 24 h of growth in the presence of dead-cell lysate (produced by sonication and filtration) in LB medium. Each point shows one gene. Dark points in the red and blue areas show significantly differentially expressed genes (false discovery rate <0.1 and fold change >2); yellow points show differentially expressed genes that are motility associated (defined here as falling under one of the following five GO terms: GO:0071973 [bacterial-type flagellum-dependent cell motility]; GO:0071978 [bacterial-type flagellum-dependent swarming motility]; GO:0044780 [bacterial-type flagellum assembly]; GO:0006935 [chemotaxis]; and GO:0044781 [bacterial-type flagellum organization]).

We next aimed to find out whether the negative effect of dead bacteria on population growth of *E. coli* in rich medium (LB) was associated with changes in gene expression in the live cell population. We performed RNA sequencing on *E. coli* populations grown with or without dead cells (sonicated-and-filtered dead-cell preparation) to identify genes involved. We found a total of 321 genes to be differentially expressed in the dead-cell treatment compared with the dead-cell-free treatment (average expression change of at least 2-fold and false discovery rate <0.1; see the ‘Materials and Methods’ section; Fig. 4, Table S2, Supporting Information). 53 of these genes were differentially expressed at more than one of the three time points we sampled, of which 32 were exclusively upregulated. Of these upregulated genes, 19 were involved in motility, chemotaxis and flagellum synthesis (Fig. 4; Table S2, Supporting Information). This was further corroborated by enrichment analysis using gene ontology (GO) categories, which showed significant enrichment of a total of 11 GO categories in the upregulated genes (Table S3, Supporting Information). Of these eleven categories, four were motility-associated, including two that were enriched at more than one time point. Individual motility genes that were upregulated included multiple genes of the *flg* (9) and *fli* (7) operons, as well as *motA*,*fliA* and *flhC*. These three genes are all transcriptional activators of motility and chemotaxis in *E. coli* (Liu and Matsumura 1995, 1996, Arnosti and Chamberlin 2006), and therefore probably contribute to the observed increased expression of other motility/chemotaxis-associated genes, which is consistent with recent work demonstrating that the presence of dead cells can affect *E. coli* swarms and increase their antibiotic resistance (Bhattacharyya *et al*. 2020). We also observed a shift in expression for several genes involved in purine (*pur* operon) and pyrimidine (c*arA* and *carB*) biosynthesis, ribose uptake systems (*rbsABCD*), and the ferric citrate (Fec) uptake system, including *fecIR*,*exbBD*,*tonB* and the *fep* operon.

### Altered expression of motility genes affects population growth and response to dead cells

**Figure 5.**
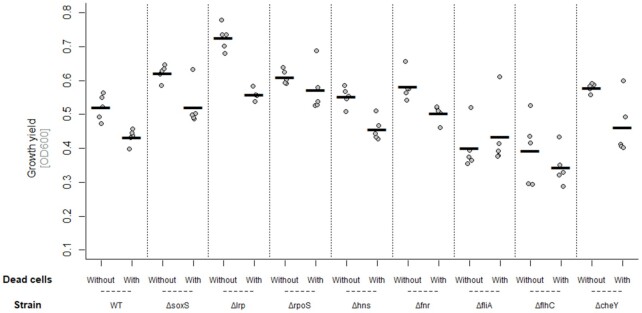
Bacterial growth (change in OD600 over 24 h) of nine single-gene deletion mutants of *E. coli* in the presence and absence of dead-cell lysate (50 µL sonicated and filtered lysate added to 100 µL culture) in LB medium. All points represent independent replicates (*n* = 5). The line shows the mean. We used a total volume of 150 µL, consisting of 1.5 µL overnight culture with 148.5 µL of dead-cell suspension. Note that dead-cell suspension was always produced from the marked wild-type MG1655 K-12 strain (see the ‘Materials and Methods’ section).

To test for further evidence that changes in gene expression identified earlier were involved in responding to dead cells, we assayed single-gene knockout strains in the presence and absence of dead cells. We did this for nine genes, focusing on those with regulatory functions and showing consistent fold changes across time points earlier, either themselves or downstream (see the ‘Materials and Methods’ section), using the same protocol and ratio of dead: live cells as earlier in our RNA sequencing experiment. We found the response to dead cells varied among different knockout strains (strain × dead cells interaction, *F*_8,72_ = 2.704, *P* = 0.01; Fig. 5). Most knockout strains showed a reduction in final population density in the presence of dead cells, as observed for the wild type here and in our other experiments. However, both the *fliA* and *flhC* knockout strain did not respond to dead cells (Welch two-sample *t*-test—*fliA*: *t* = 0.61, df = 7.05, *P* = 0.56; *flhC*: *t =* −0.96, df = 6.25, *P* = 0.37; Fig. 5), consistent with them being involved in the wild-type response to dead cells. A later repetition of this assay with the higher dead:live cell ratio used in some of our other experiments confirmed these mutants responded more weakly than the wild type to dead cells (strain × dead cells interaction, *F*_3,86_ = 4.24, *P* = 0.007; Fig. S11, Supporting Information). *flhC* is one-half of the *flhCD* dual transcription regulator, which, among other genes, controls expression of *fliA. fliA* encodes σ^28^, a minor alternative sigma factor involved in regulation of genes involved in motility, chemotaxis and flagellar synthesis (Liu and Matsumura 1995, 1996, Arnosti and Chamberlin 2006). Many genes downstream of *flhC* and *fliA* were also upregulated in our transcriptomic analysis (Fig. 4; Table S1, Supporting Information).

To further corroborate the importance of these two genes and their downstream genes, we also used overexpression from the ASKA library for *flhC* and *fliA*. These strains contain a high copy-number plasmid with the gene of interest under control of the *lacZ* promoter, inducible by IPTG. We found adding IPTG caused a significant reduction in bacterial growth for both the *flhC* and *fliA* overexpression strains (Fig. S12, Supporting Information), consistent with overexpression of these genes being costly. In contrast, adding IPTG did not change average growth of K-12 MG1655 in the same experiment (Fig. S12, Supporting Information), suggesting the observed growth reduction for the ASKA strains did not reflect a general effect of IPTG on bacterial growth. When we added dead cells, neither *flhC* nor *fliA* overexpression strains showed reduced growth (Fig. S12, Supporting Information). In the presence of IPTG, this may be explained by them already paying the cost of overexpression of these genes in both the presence and absence of dead cells. In the absence of IPTG this is more surprising, because we would expect them to show reduced growth in response to dead cells as in our other experiments. This may be explained by strain differences between the ASKA hosts K-12 AG1 and K-12 MG1655 (used in our other experiments, and showing the same reduced growth in response to dead cells as before when tested in the same block of assays as the ASKA strains; Fig. S12, Supporting Information), or by leaky expression of the plasmid-borne gene copies in the ASKA strains (Chen *et al*. 2018). Consistent with the latter, the ASKA strains reached lower stationary phase densities than K-12 MG1655 even without induction by IPTG.

### No changes in swimming motility detected in response to dead-cell treatment

We next tested whether motility behavior of *E. coli* was affected by the addition of dead-cell suspension. However, we found no consistent effect of exposure to lysed cells on swimming motility of the wild type on soft agar (Fig. S1, Supporting Information). The lack of detectable changes in swimming behavior could be because the observed overproduction of transcripts involved in chemotaxis does not translate to an altered motility phenotype, but we do not exclude that there were altered motility phenotypes not detected by this type of assay, particularly given that there are several different types of motility in *E. coli* (Garbeva and de Boer 2009, Kearns 2010), and recent evidence that exposure to dead cells induces altered swarming behavior upon antibiotic exposure (Bhattacharyya *et al*. 2020).

### No evidence of changes in biofilm formation in response to dead cells in LB medium

We hypothesized that increased biofilm production might explain both the lower population densities and the transcriptional changes observed when we treated *E. coli* with lysed cells in LB. Motility genes are known to be involved in biofilm formation by facilitating cell adherence (Pratt and Kolter 1998, Wood *et al*. 2006). Furthermore, dead cells release compounds that can be used in biofilm formation, such as eDNA, proteins and vesicles (Whitchurch 2002, Turnbull *et al*. 2016, Feng *et al*. 2018). However, addition of dead-cell lysate did not affect biofilm formation compared with the control treatment in LB, and even lowered it in M9 with glucose (Fig. S13, Supporting Information).

## Discussion

We investigated the effect of dead bacteria on live bacteria. We found that in low-nutrient environments, adding lysate prepared from dead bacteria increased the final population density of neighboring live cells, consistent with the widely held view that dead cells play a nutritional role. In contrast, we found that when bacteria were supplied with other nutrients, adding dead-cell lysate negatively affected final population density. The type of dead cells was important: we found a large effect using phage-lysed or sonicated cells, whereas cells that were killed by heat showed a marginal effect (note that the lack of effect for heat-killed cells was not due to heat lability of active ingredients; sonication prior to heating restored the negative effect on live cells). Our transcriptomic study of the response of *E. coli* to dead bacteria in LB revealed upregulation of flagellar synthesis and motility-associated genes. Knocking these genes out supported their involvement in the response to dead bacteria. In contrast, overexpression of the same genes was costly to bacteria in terms of population growth in these conditions, which probably contributes to the reduced population density we observed upon exposure to dead-cell lysate. Extensive cell lysis is common in nature, for example by phages (Weinbauer 2004), antibiotics (Lederberg 1957, Yu *et al*. 2015) or type VI secretion (Alteri and Mobley 2016), indicating such effects may apply in conditions beyond those tested here.

Together, these data show while dead cells can serve as nutrients, they can also promote other behavior in bacteria that reduces net population growth. This is in contrast to much previous work, which has often focused on the role of dead cells in nutrient recycling in starving cultures (Steinhaus and Birkeland 1939, Koch 1959, Postgate and Hunter 1963, Takano *et al*. 2017, Schink *et al*. 2019), late stationary phase and so-called GASP (growth advantage in stationary phase) phenotypes (Zambrano and Kolter 1996, Finkel and Kolter 1999, Zinser and Kolter 1999, 2001, 2004). In line with this research, we observe that *E. coli* can indeed grow on dead cells in buffer with no other available carbon source. However, when supplied with other nutrients, we see that this positive effect becomes progressively smaller when more nutrients are present, and eventually becomes negative. These different observations potentially reflect the different physiological state of *E. coli* in starvation/with no other nutrients supplied compared with that in nutrient-rich medium. Growth of *E. coli* in LB is known to be limited by depletion of utilizable carbon (Sezonov *et al*. 2007). Combined with our observation that manipulation of nutrient concentration in LB, but not population density, altered the effect of dead cells on population growth, this suggests that the total amount of available nutrients in our experiments is important in the observed response to dead cells. Induction of the stringent response via RpoS (Battesti *et al*. 2011), and the subsequent wide-ranging changes in gene expression, may be an important determinant of the ability of *E. coli* to feed off dead cells in these conditions. This is corroborated by the central role of RpoS mutations in the GASP phenotype that appear in late stationary phases, and presumably confer increased ability to use nutrients released by dead cells (Zinser and Kolter 2004). Another difference between our experiments and work with starved cultures is how cells have died (lysis vs starvation). Cause of death has been shown to influence responses to dead cells in several other systems (Herker *et al*. 2004, Kono and Rock 2008, Durand *et al*. 2011, Medina *et al*. 2020). Thus, we do not rule out that starved cells, which are commonly used to study nutrient recycling of dead-cell material, may have different effects from those observed here for lysed cells because, for example, prolonged starvation may cause cells to break down intracellular components prior to death.

A key question for future research is how the negative response to dead cells in terms of population growth in the high-nutrient environment has evolved. One possible explanation is an adaptive response that might protect bacteria from stress. Dead cells, especially dead conspecifics, may be reliable indicators of lethal stress nearby (LeRoux *et al*. 2015b, Westhoff *et al*. 2017). Recent work found evidence of such ‘necrosignaling’ in *E. coli*, by showing that swarming *E. coli* cells respond to released protein from dead clone mates, increasing their ability to withstand antibiotic stress and subsequently swarm on agar with an otherwise lethal concentration of antibiotic (Bhattacharyya *et al*. 2020). This increase in antibiotic resistance is only observed in swarming *E. coli*, and agrees with both our transcriptomic data pointing to a link between a dead-cell response and motile behavior, and our failure to observe altered swimming behavior in the presence of dead cells. Our work also goes beyond these past findings by addressing the effect of dead cells on population growth, and how this depends on local nutrient status. We speculate that the increased transcription of motility-associated genes may reflect a ‘fleeing’ response to dead cells and associated stressors. Future work testing whether the overexpression of motility-associated genes and reduced population growth we observed in the presence of dead cells confers a fitness benefit to bacteria in other types of stressful conditions will shed light on this idea. Although our results strongly implicate motility-associated genes in the observed response to dead cells, we also observed changes in gene expression for some other types of genes. Although our genetic knockouts indicated that defective motility systems ameliorated the observed effect of dead cells, we do not rule out other types of responses.

In conclusion, our results show that dead bacteria influence live bacterial cultures, including a novel, growth-suppressive effect in a high-nutrient environment. This depends critically on how the bacteria involved died (lysed or not) and which environment the live cells are growing in. The response appears to involve upregulation of motility- and chemotaxis-associated genes, although we also observed changes in expression of several other genes. Because natural environments include a diverse range of nutrient contents and lethal stressors, these results are likely relevant outside the laboratory. For example, the nutrient medium we used (LB) mainly contains amino acids as carbon source (Sezonov *et al*. 2007), thought to be a major component of the nutrients available in the gut (Alpert *et al*. 2009). Similarly, because of the huge abundance of bacteria in the gastrointestinal tract, dead cells are likely to be present in significant quantities in such environments, some of which may be lysed as in our experiments, for example due to killing by other bacteria, antibiotics or viruses. Another area where the variable effects of dead cells are likely to be important is in soil microbiology and biogeochemical nutrient cycling, as bacteria are major decomposers in such habitats. Further information about the composition of lysates with different effects on neighboring live cells, such as their contents and amounts of carbon, nitrogen, phosphorus and other micronutrients, could shed more light on how dead cells can influence growth, and how these results might differ between environments. Another key implication of our findings is in population biology, where theoretical models of microbial populations frequently assume that dead cells simply exit the model. Our results suggest instead that dead cells can have important effects by regulating population density.

## Acknowledgments

We thank Gregory Velicer, Daniel Rozen and Rolf Kümmerli for feedback on previous versions, and Yang Shen and Martin Loessner for sonication help.

## Funding

This work was partly funded by the Swiss National Science Foundation under grant 31003A_165803 to Alex Hall.

## Supplementary Material

fiac011_Supplemental_Files
