# Supplementary material for: Exposure to lysed bacteria can promote or inhibit growth of neighboring live bacteria depending on local abiotic conditions
Source: FEMS Microbiol Ecol. 2022 Feb 9;98(2):fiac011. doi: 10.1093/femsec/fiac011 (PMC8902688; doi:10.1093/femsec/fiac011)
Supplement: fiac011_Supplemental_Files [file fiac011_supplemental_files.zip › Supplemental_table_Table_S3.docx]

| GOterm | ID |
| --- | --- |
| Bacterial-type flagellum organization | GO:0044781 |
| Bacterial-type flagellum assembly | GO:0044780 |
| Bacterial-type flagellum-dependent cell motility | GO:0071973 |
| Bacterial-type flagellum-dependent swarming motility | GO:0071978 |
| Chemotaxis | GO:0006935 |
| ‘de novo’ IMP biosynthetic process | GO:0006189 |
| tricarboxylic acid cycle | GO:0006099 |
| glycine decarboxylation via glycine cleavage system | GO:0019464 |
| hydrogen sulfide biosynthetic process | GO:0070814 |
| DNA unwinding involved in DNA replication | GO:0006268 |
| tRNA wobble uridine modification | GO:0002098 |

**Table S3 All 11 GO term categories that showed a significant upregulation at at least one of the timepoints.**
