# Supplementary material for: Exposure to lysed bacteria can promote or inhibit growth of neighboring live bacteria depending on local abiotic conditions
Source: FEMS Microbiol Ecol. 2022 Feb 9;98(2):fiac011. doi: 10.1093/femsec/fiac011 (PMC8902688; doi:10.1093/femsec/fiac011)
Supplement: fiac011_Supplemental_Files [file fiac011_supplemental_files.zip › Supplemental_Data.docx]

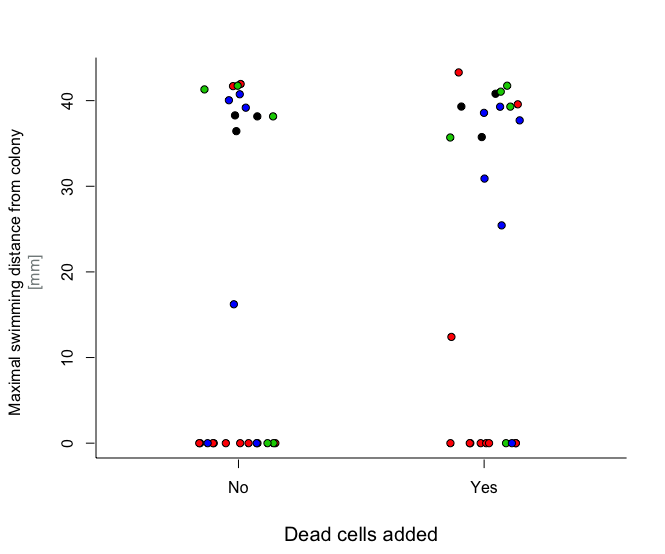


A

B>


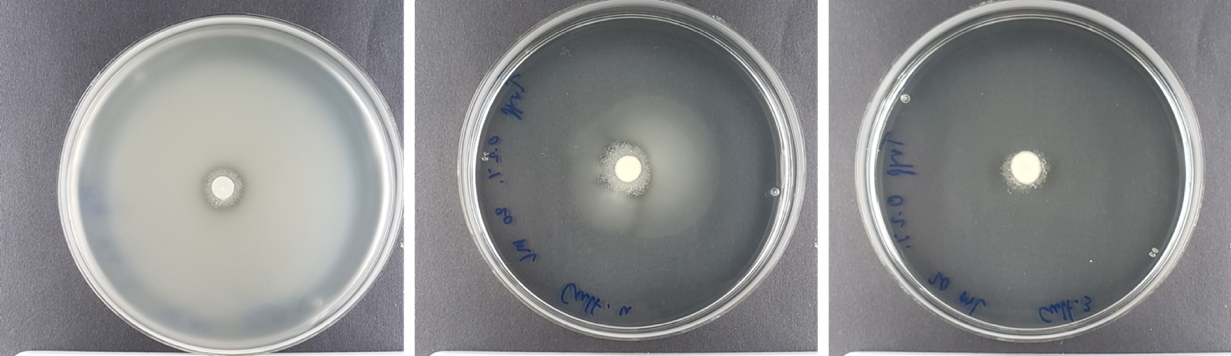


**Figure S1 No consistently visible effect of dead cells on E. coli swimming motility.** (A) Examples of motility plates showing different degrees of swimming, with maximal swimming on the left, partial swimming in the middle and no swimming on the right. (B) The effect of sonicated filtered dead cells on maximal swimming distance of E. coli on the same soft LB agar plates (0.2% agar). Each point shows one motility plate, measured in one of four different experimental blocks (different blocks shown by different colours). Maximal swimming distance on each plate is defined as the longest straight distance from the edge of the inoculated colony to the edge of the motility halo. The maximal distance possible is 45 mm (experiments were done in 90 mm petri dishes). Suspending E. coli in dead cells had no significant effect on average swimming distance here (linear mixed effects model with experimental block as a random factor, t = 0.74, p = 0.46). This is unchanged when we exclude plates that showed no swimming (maximum radius < 1 mm, which may result from plate-to-plate variation of agar consistency; linear mixed effects model with experimental block as a random factor, t = -0.61, p = 0.55). We also used an alternative protocol where we inoculated E. coli in the centre of the plate with drops of sonicated dead cells or sonicated LB (control) equally distant from the plate centre. Across 6 experimental blocks we tested 70 such plates, of which 38 showed full or partial swimming. Of these 38 plates, 2 showed an asymmetric motility halo away from dead cells, but we otherwise observed no consistent bias towards or away from dead cells.


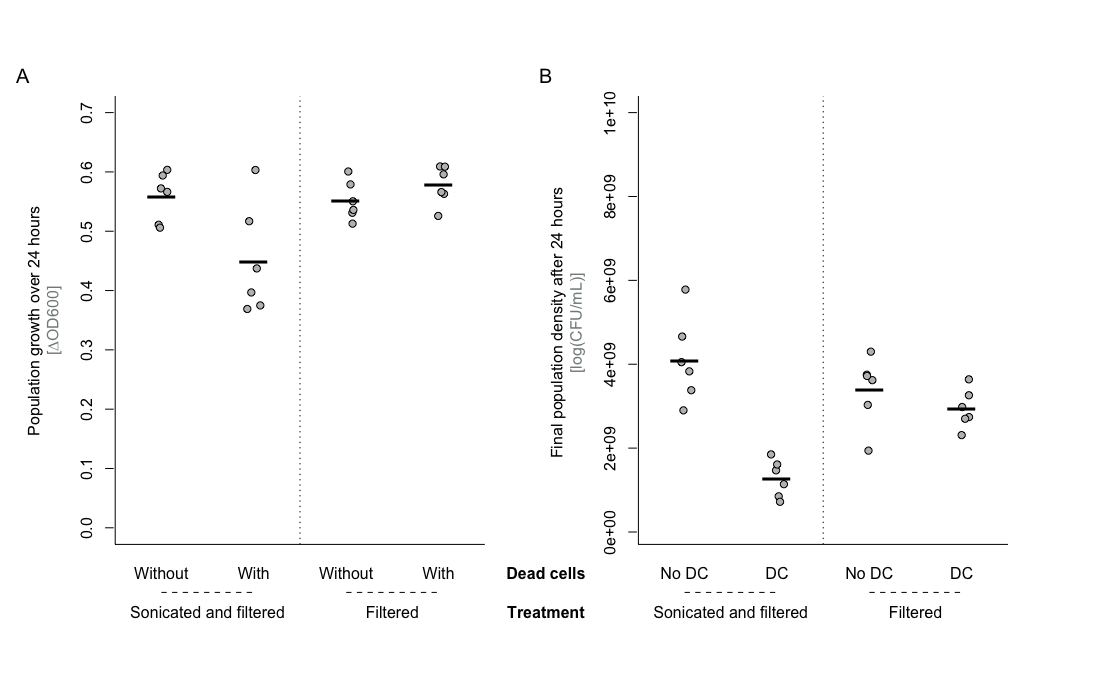


**Figure S2 Bacterial growth measured in both the change in (A) OD600 and (B) CFU/mL over 24 hours in E. coli in the presence and absence of either sonicated and filtered dead cells, or unsonicated filtered cells.** We prepared cells using either our standard protocol utilizing lysis by sonication and then filtration, or only filtration to control for the time cells were resuspended in fresh medium before filtration. The effect of dead cells was strongly affected by whether cells were sonicated or not (treatment × dead cells interaction, F_1,20_ = 8.84, p = 0.008 for OD, F_1,20_ = 22.91, p = 0.0001 for CFU/mL). All points represent independent replicates (n = 5). The line shows the mean. We used a total volume of 150 µL, consisting of 1.5 µL overnight culture with 148.5 µL of dead cell suspension.


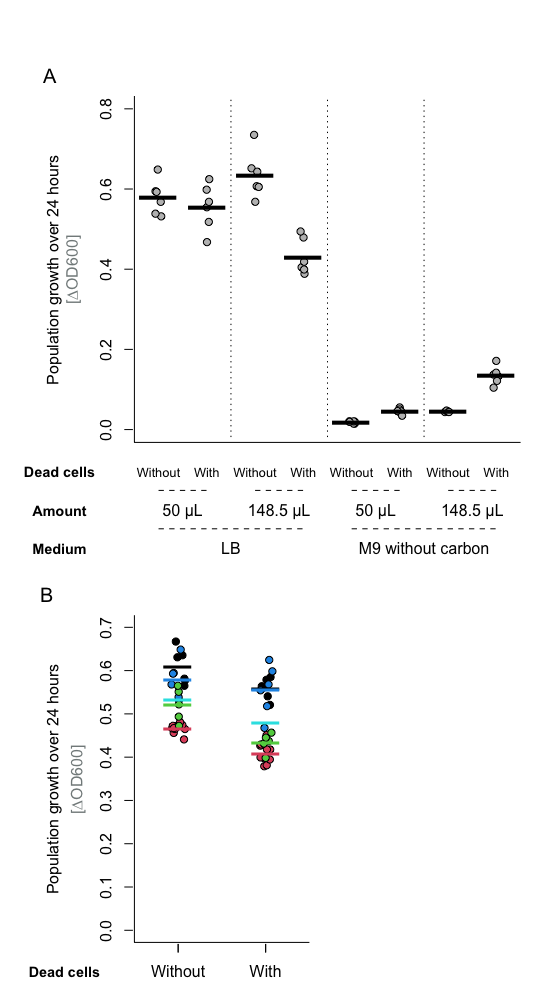


**Figure S3. Stronger effects when more dead cells are added. (A) Bacterial growth (change in optical density, OD600, over 24 hours) for *E. coli* grown in the presence/absence of different amounts of sonicated-and-filtered dead cell preparation in both nutrient-rich (LB) and minimal (M9 without carbon) medium**. We used a total culture volume of 150 µL, consisting of 1.5 µL overnight culture with either 148.5 µL of dead cell suspension, or 50 µL dead cell suspension and 98.5µL of fresh medium. All points represent independent replicates (n = 6). Each line shows the mean for one treatment group. **(B) Effect of adding 50 µL of sonicated filtered dead cells in nutrient-rich medium (LB) across multiple experimental blocks with the same experimental design**. Different experimental blocks are coded by colours, including data from the top panel and from three other experiments using the same protocol (from figures 5, S7 and S9). Despite observing a non-significant effect in the block shown in the top panel, across the various experiments using this amount of dead cells, we observed a significant effect (effect of dead cells in two-way anova including dead cells and experimental block as factors: F_1,46_ = 35.92, p < 0.0001). This effect of dead cells was consistent across the different blocks (dead cells x dataset interaction, *F*_3,46_ = 1.69, *p* = 0.18), albeit weaker than the effect observed with 148.5 µL of dead cell preparation in the top panel.

**
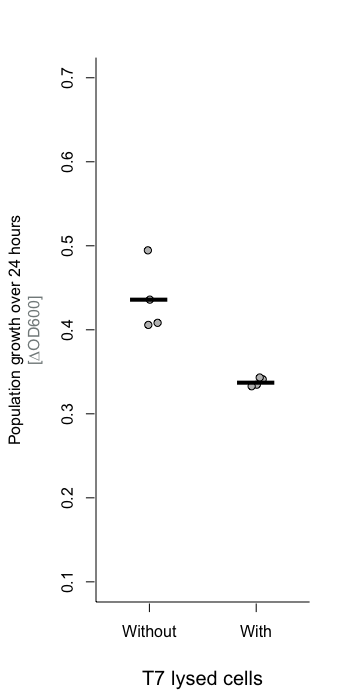
**

**Figure S4 Bacterial growth (change in OD600 over 24 hours) in *E. coli* in the presence and absence of T7-phage-lysed and filtered cells in nutrient-rich (LB) medium.** All points represent independent replicates (n = 4). The line shows the mean. We used a total volume of 150 µL, consisting of 1.5 µL overnight culture with 50 µL dead cell suspension and 98.5µL of fresh medium.


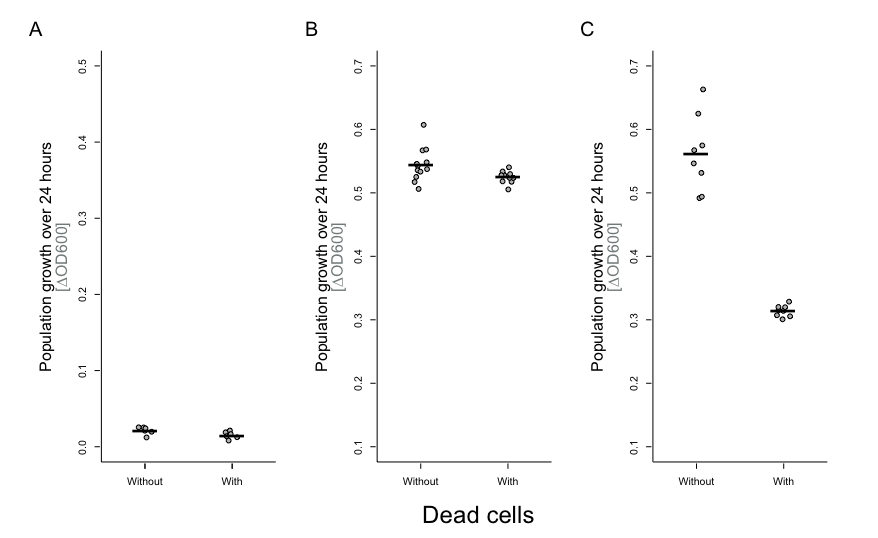


**Figure S5 Bacterial growth (change in OD600 over 24 hours) in the presence and absence of different variants of heat-killed cells in (A) minimal medium (M9, *n*=6) and (B&C) nutrient-rich medium (LB, *n*=11 and 8 respectively)**. The same type of heat-killed cells were used in (A) and (B); in (C) the dead cell preparation was first sonicated, then heat-treated. All points represent independent replicates. The line shows the mean. Note these three panels are from different experimental blocks; a direct comparison between heated and sonicated-then-heated dead cell treatments is shown in Fig. S6. We used a total culture volume of 150 µL, consisting of 1.5 µL overnight culture with 50 µL dead cell suspension and 98.5µL of fresh medium.


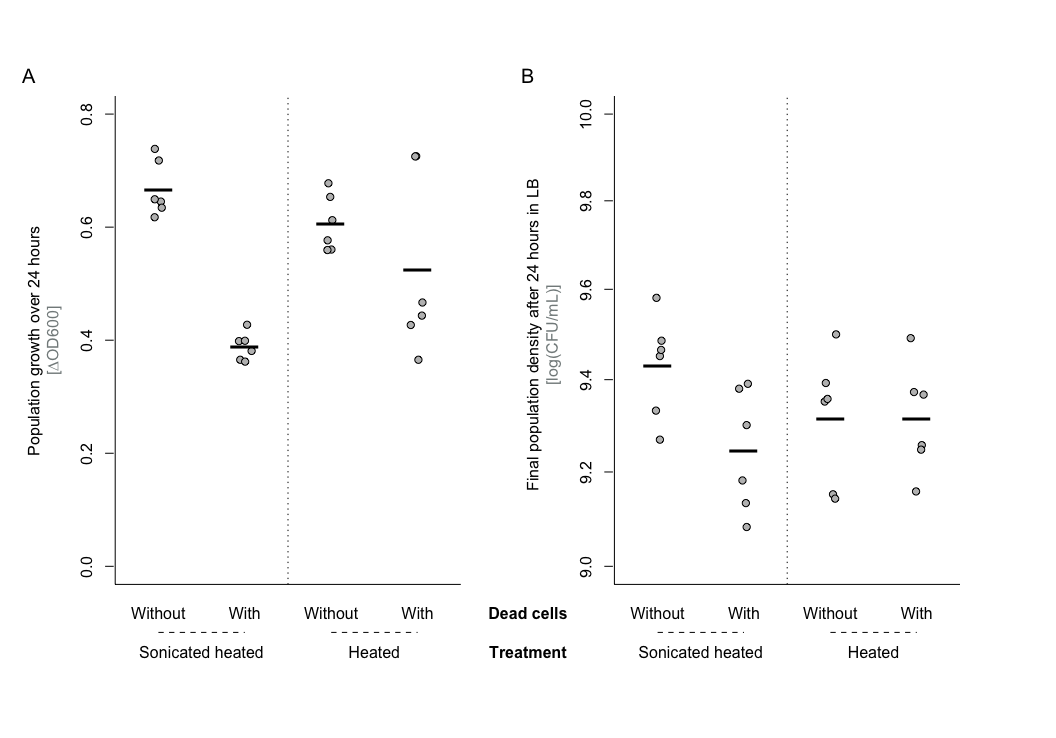


**Figure S6 Bacterial growth measured in both the change in (A) OD600 and (B) CFU/mL over 24 hours in LB in *E. coli* grown in the presence and absence of either sonicated and heated or just heated dead cells and their respective controls.** All points represent independent replicates. The line shows the mean. We used a total volume of 150 µL, consisting of 1.5 µL overnight culture with 50 µL dead cell suspension and 98.5µL of fresh medium. Note that the heated but not sonicated cells did not induce a significant effect on the population growth (Welch two-sample t-test – OD: t = -1.20, df = 5.99, p = 0.28; CFU/mL: t = -0.005, df = 9.70, p = 0.99), but the sonicated-and-heated dead cell treatment did (reported in main text).


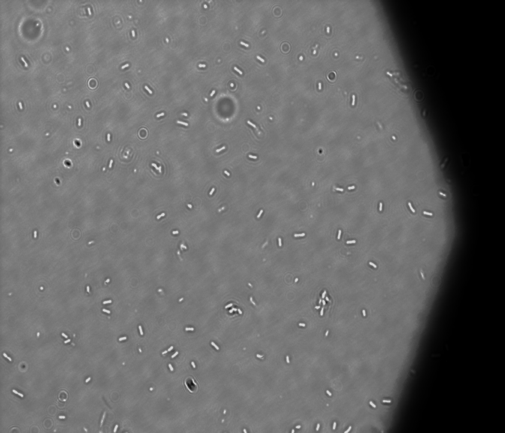

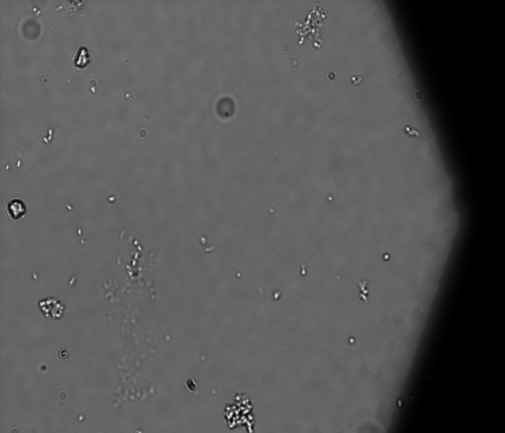


A

B


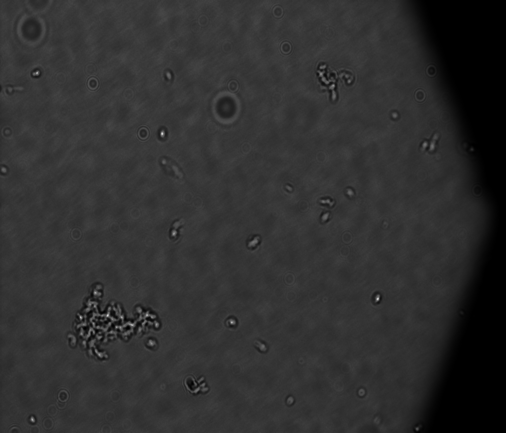

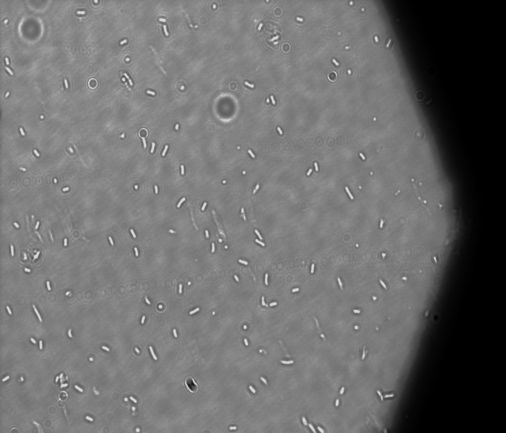

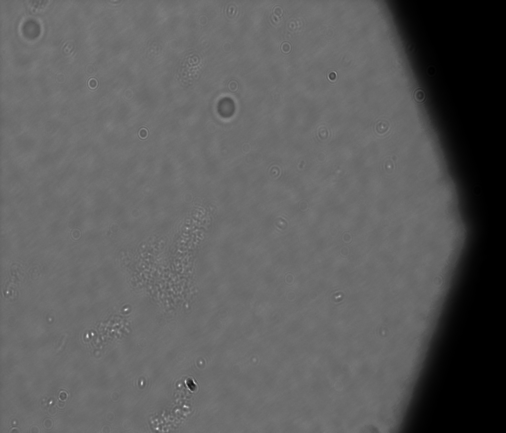

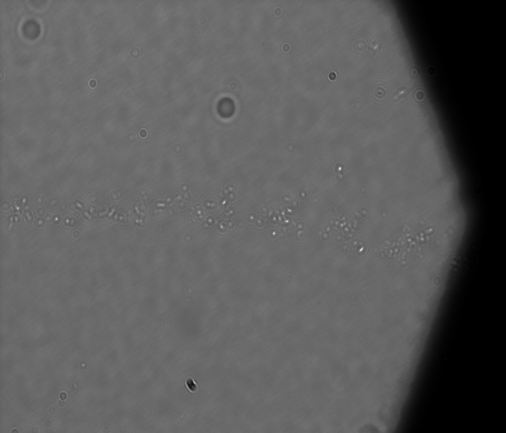

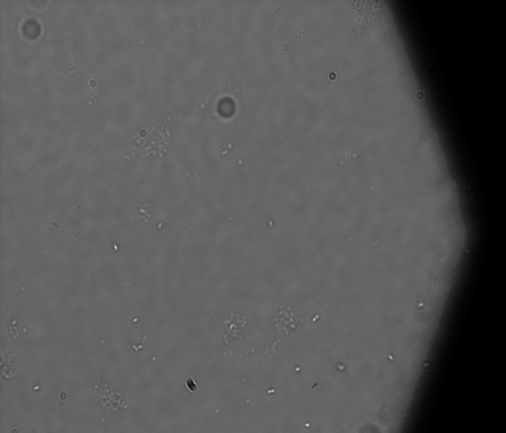

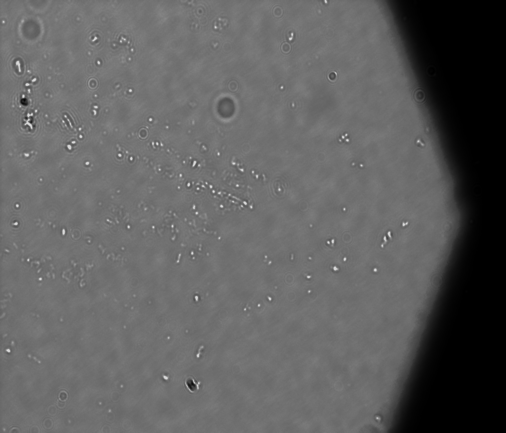


D

C

**Figure S7 Pictures of different types of live and killed cultures. (A) E. coli culture grown for 3 hours in LB medium (inoculated by 100× dilution from an overnight culture); (B) heat-killed cultures of E. coli; (C) sonicated-and-filtered cultures of E. coli; (D) phage-lysed and filtered cultures of E.coli.** Pictures were made using a Nikon Eclipse i90 microscope, using phase contrast settings on 50× magnification. The two panels in each row show independently prepared cultures.


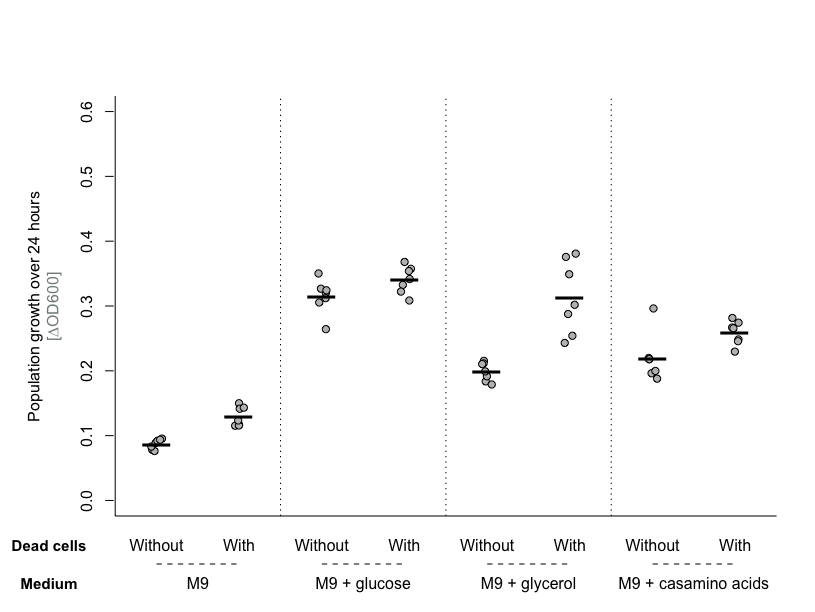


**Figure S8 Bacterial growth (change in OD600 over 24 hours) in *E. coli* in M9 medium with different carbon sources (CA= Casamino acids) in the presence and absence of sonicated filtered dead cells or control medium.** All carbon sources were added at 0.2% (*w*:*v*). All points represent independent replicates (n = 7). The line shows the mean. Note that in each of these conditions, the final bacterial density in the absence of dead cells was similar to that in relatively diluted LB treatments in Fig. 2 (OD ~0.1-0.3), where we also observed either no or weak positive responses to dead cells. Therefore, we do not exclude that populations growing to similar densities but in different abiotic conditions respond in similar ways. We used a total volume of 150 µL, consisting of 1.5 µL overnight culture with 148.5 µL of dead cell suspension.


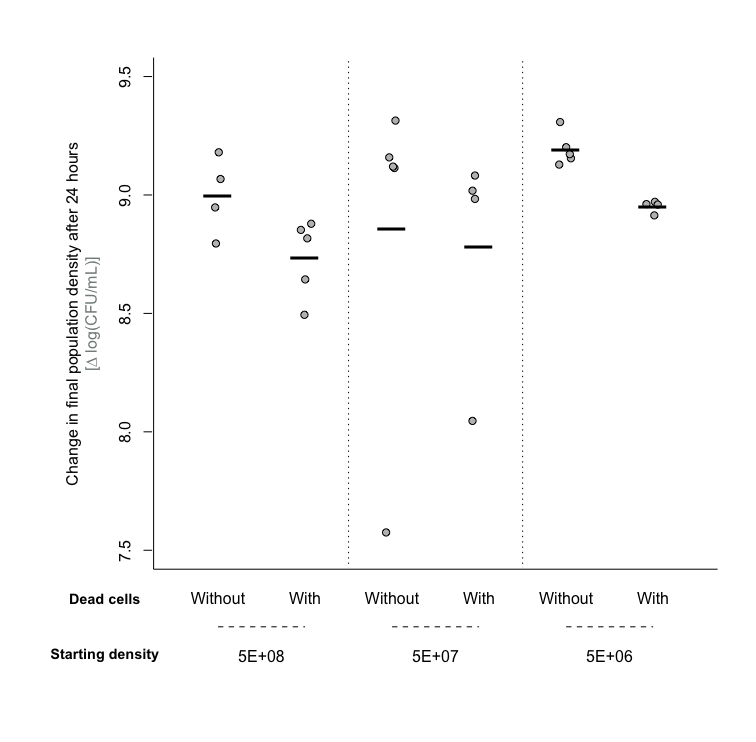


**Fig. S9 Bacterial population growth (change in CFU/mL over 24 hours in nutrient-rich medium, LB) in the presence and absence of sonicated-and-filtered dead cell preparation or control medium, using different starting densities of the live cell culture.** In all other assays, 5e+06 was used as the starting density. All points represent independent replicates (n = 5; one replicate is missing from each of the 5E+08/without, 5E+07/with and 5E+06/with treatment levels, because no colonies grew on these plates [cultures were plated at a single dilution factor]; these cultures are not plotted). Black lines show the mean. We used a total culture volume of 150 µL here, consisting of 1.5 µL overnight culture plus 148.5 µL of dead cell suspension.


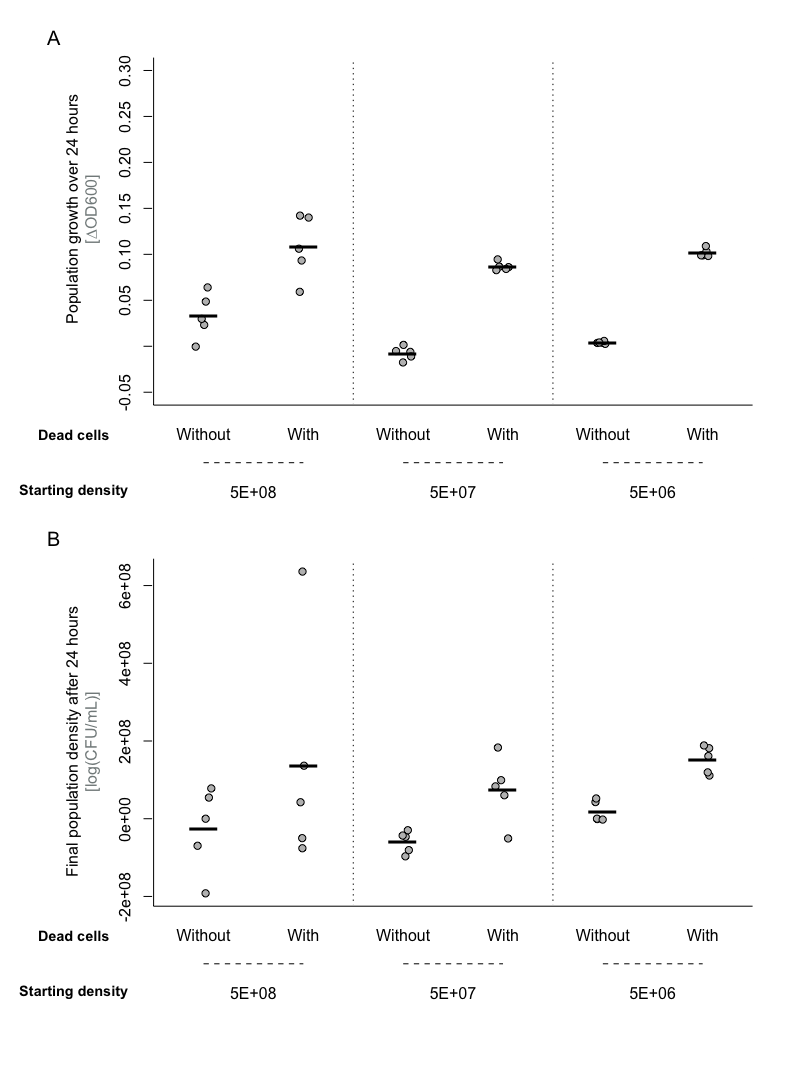


**Figure S10 Bacterial growth measured as the change in (A) OD600 or (B) CFU/mL over 24 hours in minimal medium (M9 with no additional carbon source) in the presence and absence of sonicated-and-filtered dead cell preparation using different inoculum sizes.** In all other assays, 5E+06 is used as standard inoculum. All points represent independent replicates (n = 5). The line shows the mean. We used a total volume of 150 µL, consisting of 1.5 µL overnight culture with 148.5 µL of dead cell suspension.


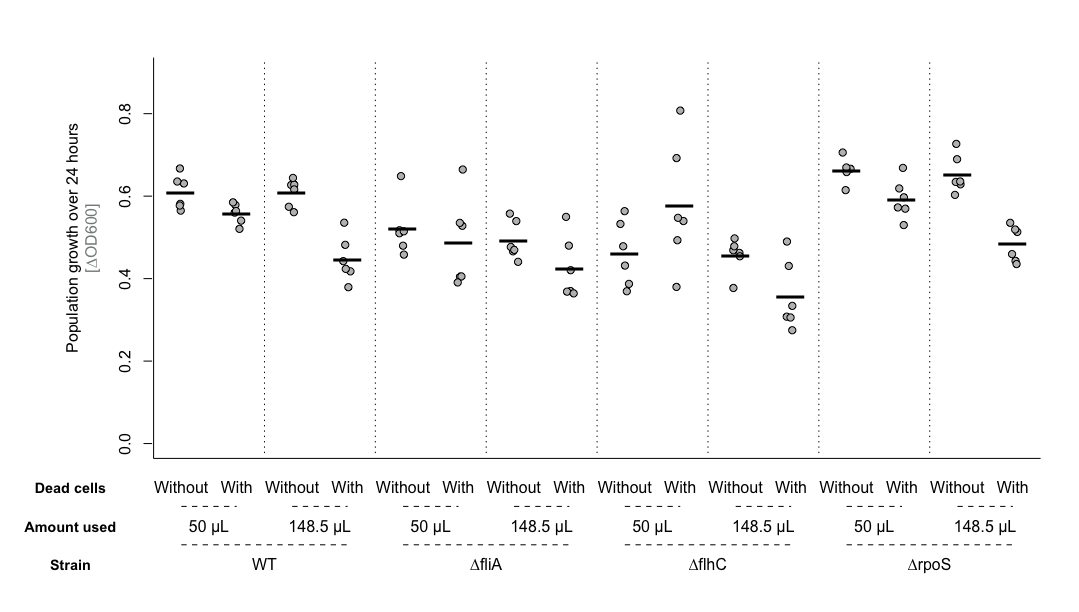


**Figure S11 Bacterial growth (change in OD600 over 24 hours) of three single-gene deletion mutants of E. coli BW 21533 in the presence and absence of sonicated-and-filtered dead cell preparation in LB medium, using different amounts of sonicated lysed cells**. We used a total volume of 150 µL, consisting of 1.5 µL overnight culture with either 148.5 µL of dead cell suspension, or 50 µL dead cell suspension and 98.5µL of fresh medium. All points represent independent replicates (n = 6). The line shows the mean.

**
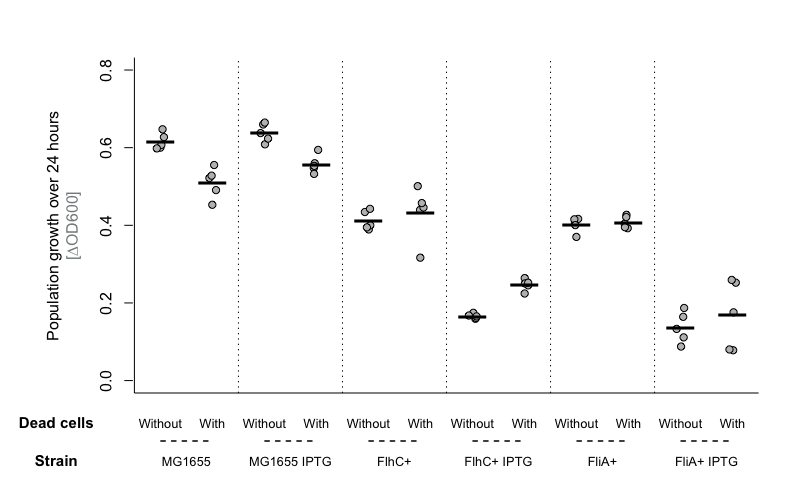
**

**Figure S12 Bacterial growth (change in OD600 over 24 hours) of ASKA overexpression strains of E. coli**  **and the wild-type in the presence and absence of sonicated-and-filtered dead cell preparation in LB medium**. Strains were either induced or not with 0.1 mM IPTG (see x-axis). All points represent independent replicates (n = 5). The line shows the mean. We used a total volume of 150 µL, consisting of 1.5 µL overnight culture with 50 µL dead cell suspension and 98.5µL of fresh medium.

**
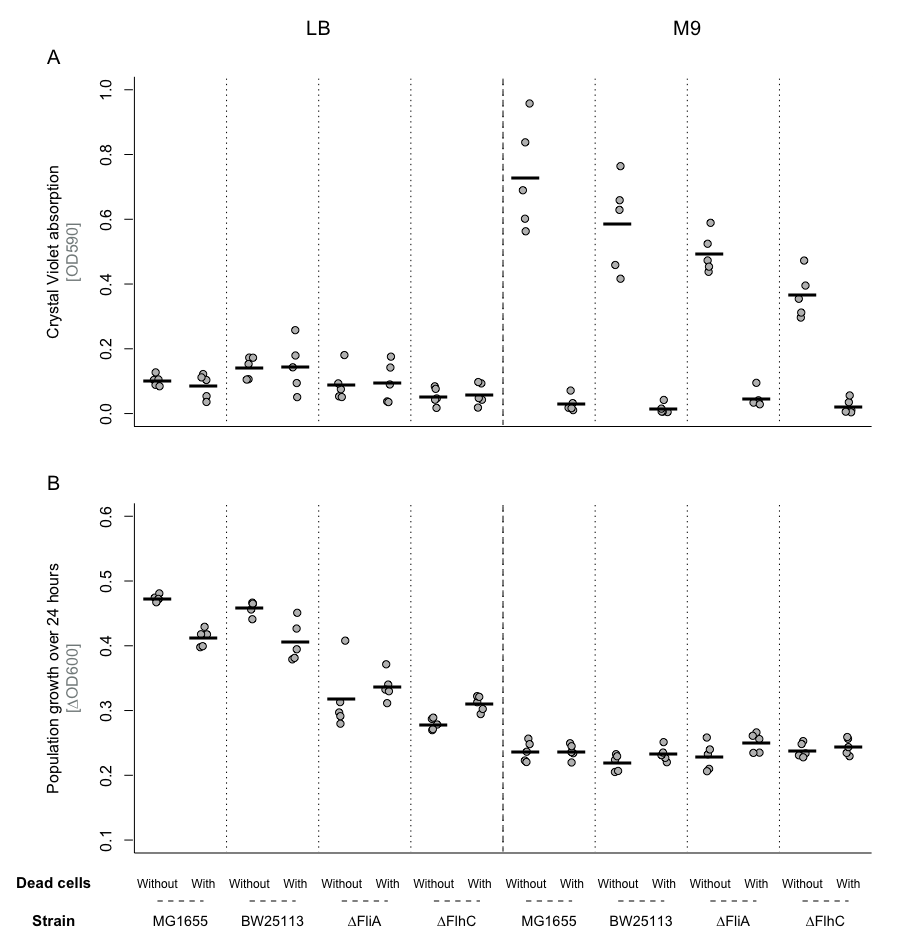
**

**Figure S13 Biofilm production and final density of E. coli MG1655, BW25113 and the fliA and flhC mutants in the presence and absence of sonicated filtered dead cells (sonicated and filtered) in LB and M9+glucose medium.** All points represent independent replicates (n = 5). The line shows the mean. Biofilm production was measured by crystal violet staining of adherent cells. We found no evidence of increased biofilm production in LB for either E. coli K12 MG1655 or BW25113 (Welch two-sample t-test - MG1655: t = 0.88, df = 5.50, p = 0.41; BW25113: t = -0.08, df = 5.42, p = 0.94), even though these cultures showed the same reduced growth yield in the presence of dead cells as we observed before (Welch two-sample t-test, t = 9.51, df = 5.07, p = p < 0.001 for MG1655; t = 3.53, df = 4.94, p = 0.017 for BW25113). We used a total volume of 150 µL, consisting of 1.5 µL overnight culture with 50 µL dead cell suspension and 98.5µL of fresh medium.
