# Supplementary material for: Exposure to lysed bacteria can promote or inhibit growth of neighboring live bacteria depending on local abiotic conditions
Source: FEMS Microbiol Ecol. 2022 Feb 9;98(2):fiac011. doi: 10.1093/femsec/fiac011 (PMC8902688; doi:10.1093/femsec/fiac011)
Supplement: fiac011_Supplemental_Files [file fiac011_supplemental_files.zip › Supplemental_table_Table_S1.docx]

| *Species* | *Strain* |
| --- | --- |
| *Escherichia coli* | K12 MG1655 |
|  | K12 MG1655 Δ*Ara* |
|  | BW 25113 |
|  | BW 25113 Δ*soxS* |
|  | BW 25113 Δ*lrp* |
|  | BW 25113 Δ*rpoS* |
|  | BW 25113 Δ*hns* |
|  | BW 25113 Δ*fnr* |
|  | BW 25113 Δ*fliA* |
|  | BW 25113 Δ*flhC* |
|  | BW 25113 Δ*cheY* |
|  | K12 AG1 JW1907 (pCA42N (*fliA*)) GFP- |
|  | K12 AG1 JW1880 (pCA42N (*flhC*)) GFP - |
|  | EcoR9 |

**Table S1. Species and strains used**
