# Supplementary material for: Exposure to lysed bacteria can promote or inhibit growth of neighboring live bacteria depending on local abiotic conditions
Source: FEMS Microbiol Ecol. 2022 Feb 9;98(2):fiac011. doi: 10.1093/femsec/fiac011 (PMC8902688; doi:10.1093/femsec/fiac011)
Supplement: fiac011_Supplemental_Files [file fiac011_supplemental_files.zip › Supplemental_table_Table_S2.docx]

**Table S3.2 All 321 genes in *E. coli* that showed > 2 fold differential expression at one or more timepoints when cultures were treated with dead *E. coli*.** Only genes with a false discovery rate < 0.1 are shown. Bold genes were > 2 fold differentially expressed at more than one timepoint. Bold and italic genes were > 2 fold differentially expressed and motility associated (defined here as falling under one of the following five GOterms: GO:0071973 (bacterial-type flagellum-dependent cell motility); GO:0071978 (bacterial-type flagellum-dependent swarming motility); GO:0044780 (bacterial-type flagellum assembly); GO:0006935 (chemotaxis); GO:0044781 (bacterial-type flagellum organization)).

| **Gene name** | **Fold-change** | | | **False Discovery Rate (FDR)** | | |
| --- | --- | --- | --- | --- | --- | --- |
|  | After 5 h | After 6.5 h | After 24h | After 5 hrs | After 6.5 h | After 24h |
| aceA |  | -2.18 |  |  | 7.59E-18 |  |
| aceB | -2.15 |  |  | 3.03E-32 |  |  |
| aceK |  | -2.31 |  |  | 9.81E-32 |  |
| acrR |  | -2.54 |  |  | 6.24E-04 |  |
| acs |  | -3.50 |  |  | 6.27E-24 |  |
| actP |  | -3.50 |  |  | 4.76E-19 |  |
| adiY |  | 2.49 |  |  | 1.16E-03 |  |
| afuC | 2.15 |  |  | 3.30E-02 |  |  |
| aldA |  | -2.16 |  |  | 1.46E-16 |  |
| aldB | -2.05 |  |  | 4.14E-07 |  |  |
| ansP |  | -2.12 |  |  | 8.14E-03 |  |
| argB |  | 2.72 |  |  | 3.73E-04 |  |
| argC |  | 2.23 |  |  | 1.49E-02 |  |
| argH |  | 2.48 |  |  | 6.90E-09 |  |
| argI |  | 4.65 |  |  | 1.52E-02 |  |
| arpA | 2.10 |  |  | 8.22E-02 |  |  |
| artJ |  | 2.10 |  |  | 1.25E-02 |  |
| aslB | 2.16 |  |  | 5.85E-02 |  |  |
| asnA | 2.89 |  |  | 1.80E-27 |  |  |
| asnB | 2.72 |  |  | 1.16E-32 |  |  |
| astA |  | -2.71 |  |  | 7.86E-05 |  |
| astD |  | -2.34 |  |  | 4.80E-05 |  |
| astE |  | -2.54 |  |  | 1.32E-02 |  |
| azuC |  | 3.21 |  |  | 1.82E-02 |  |
| bfd |  | 3.47 |  |  | 8.24E-10 |  |
| blc | -2.28 |  |  | 1.10E-04 |  |  |
| borD |  | -2.38 |  |  | 1.03E-02 |  |
| bsmA |  | -2.12 |  |  | 2.31E-05 |  |
| **cadA** | **4.20** | **2.54** |  | **4.99E-25** | **6.49E-02** |  |
| cadB | 2.08 |  |  | 3.91E-02 |  |  |
| caiF |  | -2.51 |  |  | 2.86E-03 |  |
| **carA** | **-4.09** | **-3.14** |  | **2.25E-21** | **2.11E-24** |  |
| **carB** | **-4.25** | **-5.91** |  | **3.67E-39** | **4.74E-75** |  |
| chbA |  | 2.11 |  | 4.51E-02 |  |  |
| cheA |  |  | 6.28 |  |  | 1.12E-05 |
| cheB |  |  | 4.99 |  |  | 2.49E-13 |
| cheR |  |  | 4.82 |  |  | 4.70E-04 |
| cheW |  |  | 5.86 |  |  | 3.53E-04 |
| cheY |  |  | 5.24 |  |  | 1.59E-12 |
| cheZ |  |  | 7.36 |  |  | 1.44E-16 |
| citG | 3.47 |  |  | 2.84E-04 |  |  |
| coaD |  | 2.09 |  |  | 2.90E-05 |  |
| codB |  | -3.10 |  |  | 2.00E-09 |  |
| csiE | -4.58 |  |  | 8.05E-16 |  |  |
| cspB | 2.76 |  |  | 4.27E-05 |  |  |
| cspG | 3.35 |  |  | 3.20E-04 |  |  |
| cstA |  | -3.30 |  |  | 5.66E-35 |  |
| cvpA |  | -2.40 |  |  | 3.59E-09 |  |
| cysD |  |  | 2.87 |  |  | 3.46E-02 |
| cysH |  |  | 2.23 |  |  | 5.44E-02 |
| cysP |  |  | 2.03 |  |  | 1.18E-02 |
| cysU |  | -2.10 |  |  | 7.91E-12 |  |
| cysW |  |  | 2.91 |  |  | 9.09E-04 |
| dmlA |  |  |  |  |  |  |
| **dmlA** | **-3.64** | **-4.74** |  | **2.37E-41** | **5.12E-35** |  |
| dmlR | -2.12 |  |  | 4.46E-04 |  |  |
| **dppA** | ***-2.13*** | ***-2.50*** |  | ***6.04E-23*** | ***1.26E-32*** |  |
| dppB |  | -3.92 |  |  | 2.36E-61 |  |
| dppC |  | -4.16 |  |  | 1.05E-40 |  |
| dppD |  | -4.13 |  |  | 1.65E-37 |  |
| dppF |  | -3.90 |  |  | 5.92E-37 |  |
| **entA** | **-2.30** | **-2.11** |  | **9.34E-03** | **5.91E-03** |  |
| **entB** | **-2.73** | **-2.44** |  | **2.41E-03** | **7.27E-04** |  |
| entC |  | 3.24 |  |  | 2.70E-03 |  |
| entF |  | -2.33 |  |  | 1.72E-05 |  |
| entH | -2.10 |  |  | 6.92E-02 |  |  |
| entS | 2.90 |  |  | 2.00E-04 |  |  |
| **exbB** | **2.16** | **2.57** |  | **4.86E-11** | **5.26E-27** |  |
| **exbD** | **2.16** | **2.19** |  | **3.84E-11** | **1.10E-11** |  |
| exuT | -2.01 |  |  | 3.07E-04 |  |  |
| fadA |  | -2.90 |  |  | 4.71E-06 |  |
| fadB |  | -2.52 |  |  | 1.61E-09 |  |
| fecA |  | 2.37 |  |  | 2.56E-08 |  |
| **fecI** | **2.02** | **4.03** |  | **1.37E-04** | **2.24E-15** |  |
| **fecR** | **2.24** | **4.03** |  | **5.13E-05** | **1.07E-10** |  |
| fepB |  | 2.03 |  |  | 2.16E-02 |  |
| **fepD** | **3.19** | **2.19** |  | **2.40E-03** | **3.03E-02** |  |
| **fhuF** | **2.38** | **3.54** |  | **4.13E-10** | **2.96E-08** |  |
| fimA | 2.38 |  |  |  | 2.13E-38 |  |
| **fimC** | **2.72** | **2.41** |  | **1.06E-15** | **4.68E-07** |  |
| **fimD** | **2.99** | **2.00** |  | **1.61E-22** | **3.92E-06** |  |
| **fimI** | **2.06** | **2.04** |  | **1.95E-02** | **2.70E-08** |  |
| fiu |  | 2.57 |  |  | 3.00E-03 |  |
| flgA |  |  | 2.36 |  |  | 1.92E-03 |
| **flgB** |  | ***2.17*** | ***5.86*** |  | ***2.78E-02*** | ***3.27E-17*** |
| **flgC** |  | ***2.02*** | ***6.68*** |  | ***1.98E-02*** | ***5.50E-18*** |
| **flgD** | ***2.09*** |  | ***5.50*** | ***1.89E-04*** |  | ***5.12E-09*** |
| **flgE** | ***2.06*** |  | ***4.89*** | ***2.17E-07*** |  | ***2.51E-05*** |
| **flgF** | ***2.25*** |  | ***5.58*** | ***8.29E-05*** |  | ***5.69E-28*** |
| **flgG** | ***2.30*** |  | ***4.69*** | ***3.01E-06*** |  | ***4.35E-19*** |
| **flgH** |  | ***2.12*** | ***3.78*** |  | ***1.35E-02*** | ***1.38E-11*** |
| **flgI** | ***2.05*** |  | ***4.00*** | ***2.08E-03*** |  | ***8.59E-16*** |
| flgJ |  | 4.41 |  |  |  | 2.73E-11 |
| **flgK** | ***2.02*** |  | ***6.19*** | ***1.86E-03*** |  | ***1.55E-07*** |
| flgL |  |  | 4.96 |  |  | 1.05E-04 |
| flgM |  |  | 3.34 |  |  | 2.93E-05 |
| flgN |  |  | 3.53 |  |  | 6.03E-08 |
| flhA |  |  | 3.01 |  |  | 7.93E-06 |
| **flhB** | ***2.43*** |  | ***5.31*** | ***9.01E-02*** |  | ***1.92E-06*** |
| flhC |  | 2.25 |  |  | 3.50E-06 |  |
| **fliA** | ***2.27*** |  | ***5.10*** | ***2.92E-03*** |  | ***6.52E-18*** |
| fliC |  |  | 8.06 |  |  | 3.70E-06 |
| **fliD** | ***2.17*** |  | ***4.41*** | ***3.57E-02*** |  | ***4.17E-16*** |
| fliE |  |  | 8.40 |  |  | 9.46E-06 |
| **fliF** |  | ***2.58*** | ***5.62*** |  | ***1.31E-03*** | ***7.82E-05*** |
| **fliG** | ***2.25*** |  | ***4.56*** | ***8.76E-04*** |  | ***6.76E-15*** |
| fliH |  |  | 4.76 |  |  | 2.06E-11 |
| fliI |  |  | 3.97 |  |  | 3.15E-09 |
| **fliJ** | ***2.99*** |  | ***4.11*** | ***9.73E-02*** |  | ***1.67E-03*** |
| fliK |  |  | 4.82 |  |  | 2.53E-07 |
| fliL |  |  | 5.03 |  |  | 1.13E-02 |
| **fliM** | **2.37** |  | **5.43** | **1.05E-04** |  | **1.73E-16** |
| **fliN** | **2.27** |  | **4.72** | **4.44E-02** |  | **5.64E-05** |
| fliO |  |  | 5.21 |  |  | 8.90E-04 |
| fliP |  |  | 4.20 |  |  | 4.75E-03 |
| fliS |  |  | 5.10 |  |  | 8.57E-08 |
| fliT |  |  | 3.53 |  |  | 7.01E-04 |
| fliZ |  |  | 4.44 |  |  | 8.26E-09 |
| flxA |  |  | 6.87 |  |  | 2.69E-07 |
| folK |  | 2.07 |  |  | 7.57E-06 |  |
| gadW |  | 2.79 |  |  | 5.15E-13 |  |
| gcvP |  |  | 2.01 |  |  | 5.05E-22 |
| gcvT |  |  | 2.04 |  |  | 4.28E-14 |
| gfcB | 2.81 |  |  | 6.16E-02 |  |  |
| gfcC | 3.41 |  |  | 5.32E-02 |  |  |
| **ghxP** |  | **-2.81** | **2.23** |  | **4.33E-09** | **3.33E-05** |
| glnH | -2.47 |  |  | 1.02E-04 |  |  |
| glnK | -3.34 |  |  | 2.71E-02 |  |  |
| gnsA | 2.13 |  |  | 7.56E-03 |  |  |
| **grxA** | **2.86** | **3.18** |  | **4.18E-10** | **2.30E-06** |  |
| hcr |  | -2.02 |  |  | 6.48E-03 |  |
| hdeD |  |  | -2.01 |  |  | 1.18E-02 |
| hemF |  | 2.12 |  |  | 1.34E-08 |  |
| hisC |  | -2.15 |  |  | 7.81E-07 |  |
| hisD |  | -2.05 |  |  | 1.14E-06 |  |
| iap |  | 2.57 |  |  | 5.32E-09 |  |
| ibpA |  | 2.46 |  |  | 3.29E-03 |  |
| ibpB |  | 7.00 |  |  | 5.05E-09 |  |
| ilvB |  | -2.78 |  |  | 8.56E-31 |  |
| ilvL |  | -2.75 |  |  | 3.46E-12 |  |
| ilvM |  | -2.17 |  |  | 2.98E-02 |  |
| ilvN |  | -2.65 |  |  | 1.09E-06 |  |
| ilvX |  | -6.25 |  |  | 1.76E-05 |  |
| insH1 |  | -2.44 |  |  | 1.13E-02 |  |
| insJ | 3.20 |  |  | 1.58E-12 |  |  |
| insK | 3.53 |  |  | 5.63E-12 |  |  |
| iraP | 2.47 |  |  | 1.67E-04 |  |  |
| lacY |  | -3.23 |  |  | 3.00E-03 |  |
| lacZ |  | -2.97 |  |  | 8.97E-18 |  |
| lamB |  | -3.03 |  |  | 1.06E-27 |  |
| leuA |  | -2.11 |  |  | 6.41E-03 |  |
| leuC |  | -2.03 |  |  | 8.01E-03 |  |
| lgoR |  | -2.19 |  |  | 9.02E-02 |  |
| livF |  | -2.18 |  |  | 4.42E-02 |  |
| livG |  | -2.69 |  |  | 3.00E-03 |  |
| livH |  | -2.93 |  |  | 2.84E-03 |  |
| livJ |  | -2.03 |  |  | 1.46E-03 |  |
| livM |  | -2.30 |  |  | 1.56E-03 |  |
| lsrA | -3.96 |  |  | 1.23E-04 |  |  |
| lysA |  | -20.55 |  |  | 9.26E-60 |  |
| malE |  | -3.10 |  |  | 4.49E-24 |  |
| malF |  | -5.27 |  |  | 2.36E-16 |  |
| malG |  | -3.09 |  |  | 5.32E-05 |  |
| malK |  | -5.71 |  |  | 5.84E-26 |  |
| malM |  | -3.29 |  |  | 1.29E-09 |  |
| malT | -2.41 |  |  | 2.29E-21 |  |  |
| mdtL |  | 2.35 |  |  | 1.41E-08 |  |
| melA |  | -2.04 |  |  | 7.02E-07 |  |
| melR | -2.21 |  |  | 5.74E-02 |  |  |
| metA | -2.73 |  |  | 8.25E-02 |  |  |
| metE |  | 2.52 |  |  | 5.32E-09 |  |
| mlc | -2.08 |  |  | 1.53E-04 |  |  |
| mlrA | -2.83 |  |  | 7.50E-07 |  |  |
| **motA** |  | ***2.63*** | ***6.23*** |  | ***6.00E-02*** | ***3.53E-04*** |
| motB |  |  | 6.68 |  |  | 3.04E-05 |
| mtfA | -2.13 |  |  | 7.16E-05 |  |  |
| mutM | 2.36 |  |  | 1.27E-12 |  |  |
| nadA |  |  | -2.77 |  |  | 4.83E-10 |
| nadB |  |  | -2.71 |  |  | 6.03E-07 |
| **narK** |  | **-2.11** | **-2.84** |  | **1.35E-02** | **2.92E-03** |
| narY |  |  | -2.68 |  |  | 8.59E-02 |
| nlpA |  | -2.25 |  |  | 8.42E-04 |  |
| nrdI |  | 5.17 |  |  | 2.69E-02 |  |
| nupC |  | 2.25 |  |  | 1.67E-15 |  |
| osmE |  | -2.01 |  |  | 2.62E-13 |  |
| pepE | -2.06 |  |  | 2.73E-06 |  |  |
| pgaA |  | 3.32 |  |  | 2.38E-16 |  |
| pheA |  | -5.46 |  |  | 3.02E-60 |  |
| pheM |  | 2.34 |  |  | 1.99E-02 |  |
| phnO |  | 2.01 |  |  | 4.04E-02 |  |
| phoA | 2.51 |  |  | 3.69E-08 |  |  |
| pinQ | 2.47 |  |  | 8.65E-02 |  |  |
| pinR | 3.18 |  |  | 2.19E-02 |  |  |
| pmrR |  | 2.82 |  |  | 2.20E-03 |  |
| proV |  | -2.32 |  |  | 8.20E-05 |  |
| proW |  | -2.22 |  |  | 4.87E-03 |  |
| proX |  | -2.21 |  |  | 1.69E-04 |  |
| prpB |  |  | -3.20 |  |  | 1.29E-04 |
| prpC |  |  | -2.51 |  |  | 2.90E-02 |
| purC |  |  | 2.66 |  |  | 3.26E-09 |
| purD |  |  | 5.58 |  |  | 1.17E-36 |
| purF |  |  | 2.41 |  |  | 2.49E-13 |
| purH |  |  | 3.12 |  |  | 6.89E-21 |
| purK |  |  | 2.39 |  |  | 1.12E-05 |
| **purL** |  | **-2.62** | **4.69** |  | **1.04E-25** | **7.13E-71** |
| **purM** |  | **-2.37** | **2.46** |  | **1.57E-08** | **3.15E-09** |
| **purT** | **-3.61** | **-2.35** | **4.11** | **1.60E-10** | **2.21E-04** | **6.68E-09** |
| putA |  | -2.36 |  |  | 9.95E-21 |  |
| putP |  | -2.75 |  |  | 2.07E-22 |  |
| qorA | -2.07 |  |  | 4.51E-07 |  |  |
| **ravA** | **-2.50** | **-2.06** |  | **3.61E-10** | **4.00E-07** |  |
| **rbsA** | **-13.05** | **-3.48** |  | **1.69E-93** | **9.50E-35** |  |
| **rbsB** | ***-2.27*** | ***-2.31*** |  | ***1.72E-26*** | ***3.56E-25*** |  |
| **rbsC** | **-4.46** | **-2.58** |  | **1.47E-32** | **5.61E-22** |  |
| **rbsD** | **-8.60** | **-4.31** |  | **2.08E-35** | **7.30E-37** |  |
| rbsK | -2.16 |  |  | 1.44E-16 |  |  |
| recE |  | -2.80 |  |  | 4.44E-04 |  |
| sbp |  | -2.28 |  |  | 5.29E-08 |  |
| slp | -2.07 |  |  | 8.48E-04 |  |  |
| soxR |  | 2.40 |  |  | 6.17E-02 |  |
| soxS |  | 4.40 |  |  | 1.96E-08 |  |
| sra | -2.24 |  |  | 1.56E-10 |  |  |
| srlA |  | -5.26 |  |  | 3.67E-05 |  |
| srlB |  | -11.76 |  |  | 2.69E-06 |  |
| srlD |  | -2.75 |  |  | 2.38E-05 |  |
| srlE |  | -4.76 |  |  | 2.45E-07 |  |
| sstT |  | -2.22 |  |  | 3.67E-17 |  |
| tap |  |  | 9.38 |  |  | 7.68E-10 |
| tar |  |  | 8.46 |  |  | 4.56E-04 |
| tauB | 5.56 |  |  | 3.77E-02 |  |  |
| tdcA | -4.13 |  |  | 3.82E-03 |  |  |
| tdcB |  | 4.30 |  |  | 2.12E-10 |  |
| tdcC |  | 5.92 |  |  | 1.17E-66 |  |
| tdcD |  | 5.93 |  |  | 1.41E-75 |  |
| tdcE |  | 4.92 |  |  | 2.27E-64 |  |
| tdcF |  | 5.30 |  |  | 5.03E-38 |  |
| tdcG |  | 4.56 |  |  | 5.06E-29 |  |
| thrA |  | -12.61 |  |  | 3.94E-188 |  |
| thrB |  | -7.34 |  |  | 2.68E-117 |  |
| thrC |  | -3.97 |  |  | 3.83E-72 |  |
| thrL |  | -2.85 |  |  | 2.99E-13 |  |
| tolR |  | 2.13 |  |  | 1.05E-10 |  |
| tonB |  | 2.17 |  |  | 5.05E-09 |  |
| tpr |  | -2.45 |  |  | 2.50E-02 |  |
| treB |  | -11.01 |  |  | 1.02E-162 |  |
| treC |  | -9.09 |  |  | 8.43E-167 |  |
| trxC |  | 2.49 |  |  | 8.84E-09 |  |
| tsgA | 2.08 |  |  | 4.60E-04 |  |  |
| tsr |  |  | 6.06 |  |  | 7.96E-12 |
| tsx |  | 2.03 |  |  | 6.11E-14 |  |
| ttdA |  | 2.69 |  |  | 9.85E-03 |  |
| ttdB |  | 3.20 |  |  | 2.21E-02 |  |
| ttdR |  | -2.23 |  |  | 2.13E-02 |  |
| **ttdT** | **2.80** | **4.10** |  | **4.30E-07** | **3.88E-02** |  |
| ugd |  | 2.05 |  |  | 8.51E-02 |  |
| uhpT | 2.75 |  |  | 3.48E-02 |  |  |
| uspB | -2.45 |  |  | 3.84E-06 |  |  |
| uxaA |  | -2.03 |  |  | 6.38E-09 |  |
| uxaC |  | -2.32 |  |  | 1.45E-09 |  |
| ves |  |  | 2.46 |  |  | 2.57E-02 |
| wrbA | -2.11 |  |  | 5.43E-18 |  |  |
| **xanP** | **-4.16** | **-5.29** | **3.05** | **3.26E-04** | **7.00E-18** | **8.85e-7** |
| yaaX |  | -2.50 |  |  | 3.56E-02 |  |
| yaeF |  | 2.23 |  |  | 2.72E-02 |  |
| yagE |  | -2.48 |  |  | 9.72E-05 |  |
| yagF |  | -2.01 |  |  | 2.80E-04 |  |
| **ybaE** | **-2.41** | **-2.08** |  | **5.12E-02** | **3.43E-03** |  |
| ybcH |  | -2.07 |  |  | 5.00E-02 |  |
| ybdD |  | -3.17 |  |  | 5.99E-11 |  |
| ycaD |  | 2.19 |  |  | 1.29E-03 |  |
| ycfJ |  | 2.56 |  |  | 1.61E-02 |  |
| ycgB | -2.02 |  |  | 4.43E-11 |  |  |
| ycgR |  |  | 7.46 |  |  | 4.58E-06 |
| ydcJ |  | -2.56 |  |  | 3.33E-07 |  |
| ydfU |  | 2.60 |  |  | 8.58E-02 |  |
| **ydfZ** | **-3.34** | **-2.24** |  | **1.22E-08** | **1.35E-12** |  |
| ydhC |  | 5.13 |  |  | 1.78E-45 |  |
| ydhY |  | -3.82 |  |  | 1.69E-02 |  |
| ydiE |  | 5.42 |  |  | 1.82E-03 |  |
| ydiY |  | 2.04 |  |  | 4.06E-04 |  |
| ydjX | -2.80 |  |  | 3.78E-03 |  |  |
| yeeD |  |  | 2.68 |  |  | 3.70E-03 |
| yeeR |  | -2.20 |  |  | 3.86E-04 |  |
| yehB | 2.92 |  |  | 5.86E-02 |  |  |
| yeiQ |  | 4.72 |  |  | 1.30E-42 |  |
| yfgH |  | 2.52 |  |  | 1.51E-02 |  |
| **yfgI** | **2.23** | **3.04** |  | **1.71E-02** | **5.96E-04** |  |
| yfhL |  | -2.21 |  |  | 8.98E-02 |  |
| yfiM |  | 2.06 |  |  | 8.57E-02 |  |
| ygbM |  | 2.63 |  |  | 7.67E-02 |  |
| ygcS | 2.97 |  |  | 8.43E-02 |  |  |
| ygdI |  | -2.15 |  |  | 6.22E-05 |  |
| **ygfI** | **3.13** | **2.98** |  | **2.01E-09** | **1.70E-04** |  |
| yhbU | -2.80 |  |  | 6.29E-09 |  |  |
| yhbV | -2.11 |  |  | 3.91E-05 |  |  |
| yhcC | -4.24 |  |  | 3.29E-04 |  |  |
| yhcN |  | 2.09 |  |  | 2.14E-03 |  |
| yhjE |  | -2.36 |  |  | 3.14E-02 |  |
| yhjG |  |  | 2.16 |  |  | 1.36E-06 |
| yhjH |  | 3.43 | 9.32 |  | 4.71E-02 | 3.25E-15 |
| yhjX |  | 2.08 |  |  | 8.12E-02 |  |
| yicG |  | 5.55 |  |  | 1.75E-11 |  |
| yidZ |  | 2.00 |  |  | 9.72E-05 |  |
| yjcB | 3.99 |  |  | 1.27E-12 |  |  |
| yjcH |  | -4.62 |  |  | 1.03E-08 |  |
| yjcZ |  |  | 3.78 |  |  | 3.88E-07 |
| yjeV |  |  | -2.10 |  |  | 7.17E-02 |
| **yjfN** | **-2.25** | **-2.49** |  | **4.99E-02** | **2.02E-02** |  |
| ymcE | 3.50 |  |  | 9.51E-13 |  |  |
| ymdF | -2.00 |  |  | 3.59E-02 |  |  |
| yncD |  | 2.22 |  |  | 8.27E-06 |  |
| yncG |  |  | -2.47 |  |  | 2.92E-03 |
| ynfE |  | -2.04 |  |  | 5.32E-09 |  |
| **ynfK** | **-4.12** | **-2.46** |  | **4.80E-06** | **1.12E-02** |  |
| yohC |  | -2.58 |  |  | 2.25E-03 |  |
| yohJ |  |  | -2.13 |  |  | 7.63E-04 |
| yohK | -2.28 |  |  | 1.10E-04 |  |  |
| yohO |  | 2.01 |  |  | 3.55E-02 |  |
| yojI |  | 2.32 |  |  | 1.85E-07 |  |
| yqaE | -2.01 |  |  | 4.63E-02 |  |  |
| zraP |  | 4.44 |  |  | 2.78E-20 |  |
